# Gene transfer of master autophagy regulator TFEB results in clearance of toxic protein and correction of hepatic disease in alpha-1-anti-trypsin deficiency

**DOI:** 10.1002/emmm.201202046

**Published:** 2013-02-04

**Authors:** Nunzia Pastore, Keith Blomenkamp, Fabio Annunziata, Pasquale Piccolo, Pratibha Mithbaokar, Rosa Maria Sepe, Francesco Vetrini, Donna Palmer, Philip Ng, Elena Polishchuk, Simona Iacobacci, Roman Polishchuk, Jeffrey Teckman, Andrea Ballabio, Nicola Brunetti-Pierri

**Affiliations:** 1 Telethon Institute of Genetics and MedicineNaples, Italy; 2 Department of Pediatrics, Saint Louis University School of Medicine, Cardinal Glennon Children's Medical CenterSaint Louis, MO, USA; 3 Department of Molecular and Human Genetics, Baylor College of MedicineHouston, TX, USA; 4 Jan and Dan Duncan Neurological Research Institute, Texas Children's HospitalHouston, TX, USA; 5 Department of Pediatrics, Federico II UniversityNaples, Italy

**Keywords:** alpha-1-anti-trypsin, autophagy, gene therapy, helper-dependent adenoviral vector, TFEB

## Abstract

Alpha-1-anti-trypsin deficiency is the most common genetic cause of liver disease in children and liver transplantation is currently the only available treatment. Enhancement of liver autophagy increases degradation of mutant, hepatotoxic alpha-1-anti-trypsin (ATZ). We investigated the therapeutic potential of liver-directed gene transfer of transcription factor EB (TFEB), a master gene that regulates lysosomal function and autophagy, in PiZ transgenic mice, recapitulating the human hepatic disease. Hepatocyte TFEB gene transfer resulted in dramatic reduction of hepatic ATZ, liver apoptosis and fibrosis, which are key features of alpha-1-anti-trypsin deficiency. Correction of the liver phenotype resulted from increased ATZ polymer degradation mediated by enhancement of autophagy flux and reduced ATZ monomer by decreased hepatic NFκB activation and IL-6 that drives ATZ gene expression. In conclusion, TFEB gene transfer is a novel strategy for treatment of liver disease of alpha-1-anti-trypsin deficiency. This study may pave the way towards applications of TFEB gene transfer for treatment of a wide spectrum of human disorders due to intracellular accumulation of toxic proteins.

## INTRODUCTION

Alpha-1-anti-trypsin (AAT) deficiency is the most common genetic cause of liver disease in children (Perlmutter, 2009; Sveger, 1988). It is also responsible for chronic liver disease and hepatocellular carcinoma in adults (Eriksson et al, 1986; Piitulainen et al, 2005). AAT is a member of the serine protease inhibitor (SERPIN) superfamily of structurally conserved proteins that inhibit serine proteases. AAT is synthesized in the liver and released into plasma, where it is the most abundant circulating protease inhibitor. AAT deficiency has considerable allelic heterogeneity, with more than 70 variants reported (PI types). Nevertheless, the great majority of individuals with AAT deficiency are homozygotes for the PI*Z allele while non-Z deficiency alleles are found at very low frequencies (Cox & Billingsley, 1989). The PI*Z allele encodes for a mutant AAT protein (ATZ) carrying a missense mutation (lysine for glutamate at amino acid position 342) that alters protein folding. As a result of defective folding, ATZ is prone to polymerize and aggregate in the endoplasmic reticulum (ER) of hepatocytes, causing liver injury by a gain-of-toxic mechanism. The large ATZ polymers retained in the ER account for the intrahepatocytic globules that are pathological hallmarks of the disease. The majority of PI*Z homozygous children with AAT deficiency identified through newborn screening have abnormal liver function tests during their first year of life, approximately 10% of them have prolonged jaundice, and about 2% develop liver failure (Sveger, 1976, 1988). The only curative treatment available for children with hepatic failure is liver transplantation.

Recent findings have shown that stimulation of autophagy may reduce accumulation of hepatotoxic ATZ (Hidvegi et al, 2010; Kaushal et al, 2010). Transcription factor EB (TFEB) is a master gene that regulates the number and function of lysosomes and autophagy (Medina et al, 2011; Sardiello et al, 2009; Settembre et al, 2011). In the present study, we have investigated whether hepatic gene transfer of TFEB promotes clearance of hepatotoxic ATZ. To achieve efficient *in vivo* hepatocyte gene transfer, we used helper-dependent adenoviral (HDAd) vectors, which are devoid of all viral coding sequences and are promising non-integrating vectors for liver-directed gene therapy because they have a large cloning capacity, can efficiently transduce hepatocytes, and result in long-term transgene expression without chronic toxicity (Brunetti-Pierri & Ng, 2011). Several studies have shown efficient and long-term phenotypic correction in small and large animal models of genetic disorders by HDAd vectors (Brunetti-Pierri et al, 2005a, 2006, 2009a, 2012; Brunetti-Pierri & Ng, 2009; Kim et al, 2001; Toietta et al, 2005). The HDAd vector genome remains episomal in the nuclei of transduced cells (Ross et al, 2009) and because of its non-integrating nature, it is not associated to an increased risk of insertional carcinogenesis (Stephen et al, 2010). So far, clinical applications of HDAd have been hampered by an acute toxic response mediated by the vector capsid proteins in a dose-dependent manner (Brunetti-Pierri et al, 2004). Nevertheless, strategies are under investigation to overcome this obstacle and recently, a minimally invasive and clinically attractive method for safe and efficient delivery of HDAd vectors to the liver has been developed in preclinical large animal models (Brunetti-Pierri et al, 2007, 2009b, 2012).

## RESULTS

**Figure 1:**
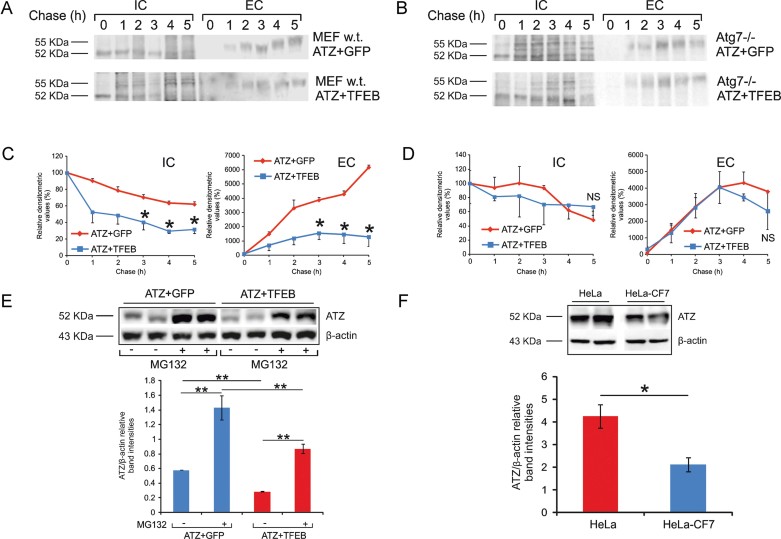
TFEB induced autophagy dependent ATZ clearance *in vitro* **A–D.** Pulse and chase analysis of ATZ in cell lysate (IC) and medium (EC) of wild-type (**A**) and Atg7^−/−^ (**B**) MEF transiently co-transfected with either ATZ plus GFP expressing plasmids or ATZ plus TFEB expressing plasmids. The corresponding densitometric analyses from two independent experiments are shown in (**C**) and (**D**). The 52 kDa bands correspond to ATZ precursor while mature ATZ is 55 kDa. (**A,C**) In wild-type MEF, the intracellular, newly synthesized 52 kDa ATZ decreased more rapidly in TFEB transfected cells compared to GFP transfected cells. In media, mature 55 kDa ATZ was secreted in greater amounts in GFP transfected cells compared to TFEB transfected cells. (**B,D**) Abrogation of autophagy in Atg7^−/−^ MEF abolished TFEB-mediated intracellular clearance of ATZ and secretion of ATZ in media was not affected.**E.** MEF transiently co-transfected with either ATZ plus GFP expressing plasmids or ATZ plus TFEB expressing plasmids were incubated with proteasome inhibitor MG132 or with DMSO for 6 h. Upon MG132 treatment, steady state levels of 52 kDa ATZ band increased in both cells transfected with ATZ plus GFP and with ATZ plus TFEB expressing plasmids. Quantification of band intensities showed a 48% reduction of ATZ in cells transfected with ATZ plus TFEB compared to cells transfected with ATZ plus GFP, in the absence of the proteasome inhibitor. In MG132 treated cells, a 61% decrease of ATZ was detected in cells transfected with ATZ plus TFEB compared to cells transfected with ATZ plus GFP.**F.** HeLa cells stably over-expressing TFEB (HeLa-CF7) accumulated less ATZ intracellularly at 24 h post-transfection with a plasmid expressing ATZ. Two independent samples from HeLa and HeLa-CF7 cells are shown. Averages ± standard deviations are shown. *p < 0.05. **p < 0.01. h, hours; NS, not statistically significant.

To investigate the efficacy of TFEB-mediated enhancement of lysosomal degradation and autophagy on ATZ clearance, we have co-transfected mouse embryonic fibroblasts (MEF) with a plasmid that expresses TFEB under the control of the CMV promoter and with a plasmid expressing the ATZ under the CMV promoter. Transfected cells were subjected to a pulse-chase radiolabelling with ^35^S-labelled Cys and Met and the resulting cell lysates and corresponding media were analysed by immunoprecipitation followed by SDS–PAGE analysis. This study showed that intracellular 52 kDa ATZ decreased more rapidly in TFEB-transfected cells compared to control cells transfected with a plasmid expressing the green fluorescent protein (GFP; Fig 1A and C). The reduction of intracellular ATZ was associated with decreased mature 55 kDa ATZ in media of TFEB transfected cells compared to control cells (Fig 1A and C). TFEB-mediated increase of ATZ clearance was not observed in Atg7^−/−^ MEFs (Komatsu et al, 2005), thus showing that functioning autophagy is needed for TFEB-mediated increase of ATZ clearance (Fig 1B and D). Treatment of MEF with proteasome inhibitor MG132 resulted in similar increase of steady state levels of intracellular ATZ in both GFP- and TFEB-transfected cells; thus TFEB does not appear to increase proteasomal degradation of ATZ (Fig 1E). ATZ protein was reduced in HeLa cells stably overexpressing TFEB (HeLa-CF7 cell line; Sardiello et al, 2009) compared to control HeLa cells (Fig 1F).

**Figure 2:**
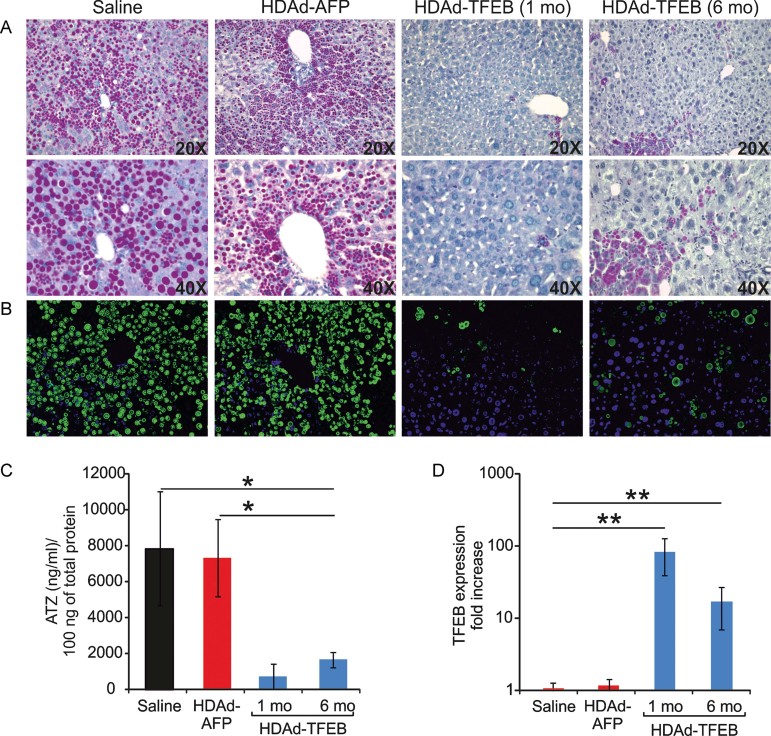
TFEB gene transfer in PiZ mice promoted hepatic ATZ clearance PAS staining was markedly reduced in livers from PiZ mice at 1 month (1 mo) and 6 months (6 mo) after the injection of HDAd-TFEB compared to saline or HDAd-AFP injected mice (magnifications 20× and 40×).ATZ immunofluorescence of livers from HDAd-TFEB injected mice showed a marked reduction of ATZ globules compared to controls at 1 and 6 months post-injection (magnification 20×). Representative images from experimental groups including at least *n* = 5 mice per group are shown.ELISA for ATZ on hepatic extracts showed a statistically significant reduction of ATZ in HDAd-TFEB injected mice at 1 and 6 months post-injection compared to HDAd-AFP and saline injected controls (at least *n* = 5 per group).TFEB real time PCR on liver mRNA showed TFEB expression as fold increase over saline at 1 and 6 months post-injection in HDAd-TFEB injected mice (at least *n* = 5 per group). Averages ± standard deviations are shown. *p < 0.05.

We next generated an HDAd vector that expresses the human TFEB cDNA under the control of a liver-specific phosphoenolpyruvate carboxykinase (PEPCK) promoter and a liver-specific enhancer (Brunetti-Pierri et al, 2005b; HDAd-TFEB; Supporting Information Fig S1) to investigate *in vivo* the therapeutic potential of TFEB gene transfer for treatment of the liver disease of AAT deficiency. We evaluated the efficacy of TFEB hepatic gene transfer in the PiZ mouse model, a transgenic mouse that expresses the human ATZ gene under the control of its endogenous regulatory regions (Carlson et al, 1988, 1989). PiZ mice recapitulate the features of liver disease observed in humans, *i.e.* intrahepatocytic ATZ-containing globules, inflammation/regenerative activity and fibrosis (Hidvegi et al, 2010). We injected 3-month-old PiZ mice (at least *n* = 5 for each group) intravenously with the HDAd-TFEB vector at the dose of 1 × 10^13^ vp/kg. Control PiZ mice were injected with either saline or with 1 × 10^13^ vp/kg of a HDAd vector that expresses the unrelated, non-immunogenic, non-toxic alpha-fetoprotein (AFP) reporter gene under the control of the same expression cassette and within the same vector backbone (Brunetti-Pierri et al, 2006; Supporting Information Fig S1) of the HDAd-TFEB vector (HDAd-AFP). No changes in appearance, behavior and body weight (Supporting Information Fig S2) were noted in PiZ mice injected with HDAd-TFEB compared to HDAd-AFP or saline injected mice for up to 6 months post-injection. The livers of PiZ mice injected with HDAd-TFEB showed at 4 weeks post-injection a dramatic reduction of both ATZ accumulation and ATZ-containing globules by periodic acid-Schiff (PAS) staining and immunofluorescence, respectively, compared to saline or HDAd-AFP injected mice (Fig 2A and B). The reduction of ATZ protein levels was confirmed by ELISA (Fig 2C) and by Western blot (Supporting Information Fig S3A) on hepatic protein extracts. The effect of HDAd-TFEB was specific and HDAd-AFP had no effect on hepatic ATZ levels (Fig 2A–C). PiZ mice were injected at 3 months of age, when a significant hepatic accumulation of ATZ is already established, as previously shown by PAS staining and ATZ immunostaining (Lindblad et al, 2007; Teckman et al, 2002) and confirmed by us (Supporting Information Fig S3B). Given the significant reduction in hepatotoxic ATZ achieved in HDAd-TFEB injected mice (Fig. 2), these results suggest that liver expression of TFEB was effective in the removal of preformed ATZ inclusions. Although slightly increased, at 6 months post-injection livers of HDAd-TFEB injected mice continued to show a clear reduction in PAS staining and ATZ protein compared to control mice (Fig 2A–C). Sustained correction of ATZ accumulation is consistent with long-term expression of TFEB, which was still detected at 6 months post-injection by real time PCR on hepatic mRNA (Fig 2D).

**Figure 3:**
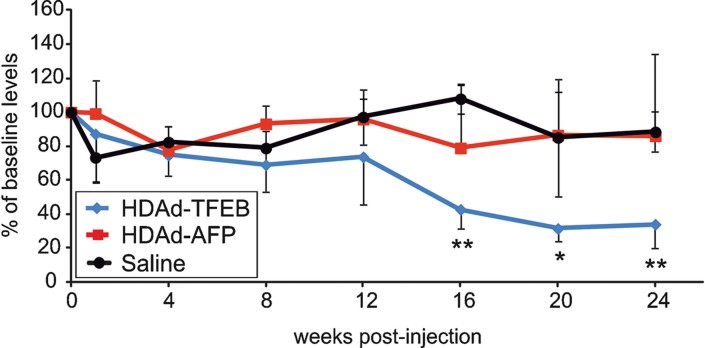
Serum ATZ is reduced in HDAd-TFEB injected mice Serum levels of human ATZ were determined by ELISA in PiZ mice injected with HDAd-TFEB and in control animals injected with saline or HDAd-AFP (at least *n* = 3 per group). Averages ± standard deviations are shown. *p < 0.05; **p < 0.01.

We observed a gradual decline in serum ATZ in PiZ mice injected with HDAd-TFEB and, at 16 weeks post-injection, the reduction of serum ATZ in these animals was approximately 43% of pre-injection levels. From 16 to 24 weeks post-injection, the amount of serum ATZ in HDAd-TFEB injected mice was significantly lower than HDAd-AFP and saline-injected mice (*p* < 0.05; Fig 3).

To rule out toxicity from TFEB hepatic gene transfer, we determined transaminases and levels of hepatocyte-specific mRNA in livers from mice injected with either HDAd-TFEB or control HDAd-AFP vector. In both experimental groups, alanine aminotransferase (ALT) and aspartate aminotransferase (AST) were found to be within the normal range or slightly above it at baseline and at various time points post-injection (Supporting Information Fig S4). No statistical significant differences were found between HDAd-TFEB and HDAd-AFP injected mice for the mRNA levels of selected hepatocyte-specific genes, including mouse transferrin receptor (mTFRC), mouse ornithine carbamoyltransferase (mOTC) and mouse factor IX (mFIX) (Supporting Information Fig S5). In summary, no clear evidence of liver toxicity was detected in HDAd-TFEB injected PiZ mice up to 6 months post-injection.

**Figure 4:**
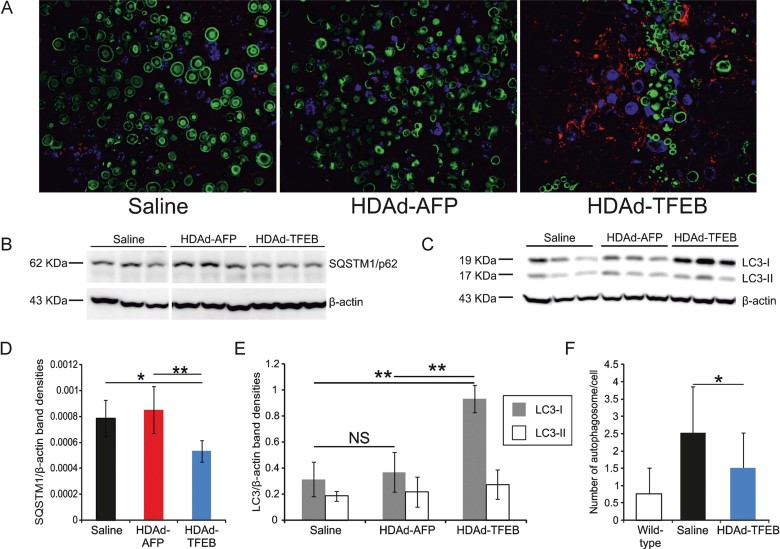
Increased liver autophagy following hepatic TFEB gene transfer in PiZ mice **A.** Co-staining of liver specimens for LAMP-1 (red) and ATZ (green) showed an increase in LAMP-1 staining at 1 month post-injection in HDAd-TFEB injected PiZ mice. For HDAd-TFEB injected livers the selected field of view shows a region of increased LAMP-1 signal and absent ATZ, surrounding an area of increased ATZ staining (magnification 63×).**B,C.** Western blots for SQTSTM1/p62 and LC3 showed a decrease in SQTSTM1/p62 and an increase in LC3-I in PiZ mice injected with HDAd-TFEB compared to controls. Representative bands from 3 mice for each treatment group are shown.**D,E.** The graphs show densitometric quantification of *n* = 5 mice from each treatment group. Quantification of band intensities was normalized for β-actin.**F.** Quantification of autophagosome number by EM in wild-type C56BL/6 mice, and saline or HDAd-TFEB injected PiZ mice (*n* = 32 cells analysed). Averages ± standard deviations are shown. *p < 0.05; **p < 0.01.

Consistent with TFEB-mediated activation of lysosome biogenesis (Medina et al, 2011; Sardiello et al, 2009), high levels of LAMP-1 were observed in livers of HDAd-TFEB injected animals (Fig 4A). Interestingly, a negative correlation was noted between ATZ and LAMP-1 immunostaining signals and the few areas positive for ATZ signals showed less increase in LAMP-1 expression (Fig 4A). SQSTM1/p62 is incorporated into completed autophagosome and are degraded in autolysosomes, thus serving as a read-out of autophagic degradation (Klionsky et al, 2012; Pankiv et al, 2007). The SQSTM1/p62 was reduced in the livers of mice injected with HDAd-TFEB compared to saline or HDAd-AFP injected mice (Fig 4B and D). An increase in LC3-I levels was also observed in livers of mice injected with HDAd-TFEB, compared to saline or HDAd-AFP injected mice (Fig 4C and E). Despite evidence of autophagy increase (enhanced SQSTM1/p62 degradation and transformation of ATZ inclusions into autophagosomes, see Fig 7 and 8), the overall number of autophagosomes counted in thin sections decreased upon TFEB treatment (Fig 4F). This observation suggests TFEB gene transfer stimulates fusion of autophagosomes with lysosomes (which are also increased in number, see Fig 4A), and thus it accelerates the rate of autophagy flux. Increased autophagy flux may also explain the apparently unchanged levels of LC3-II that may reflect increased LC3-II consumption in the autophagosome–lysosome fusion process (Fig 4C and E). Taken together, these results showed that hepatic gene transfer of TFEB enhances autophagy and lysosome biogenesis in the liver and reduces accumulation of ATZ in livers of PiZ mice.

**Figure 5:**
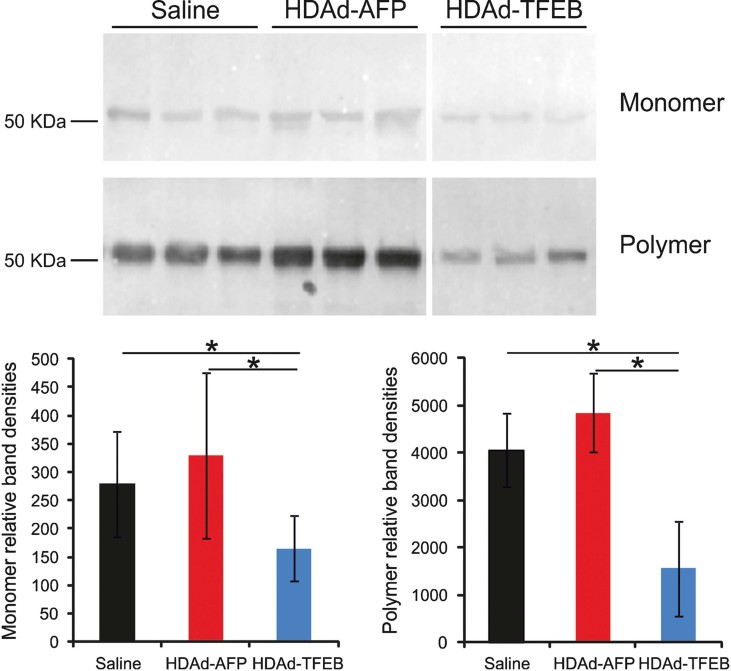
TFEB gene transfer reduced hepatic ATZ monomer and polymer in PiZ mice Representative bands from three PiZ mice for each treatment group are shown. The graph shows densitometric quantification of *n* = 5 PiZ mice from each treatment group. A statistically significant decrease in both ATZ monomer and polymer was observed in HDAd-TFEB injected mouse livers compared to either saline or HDAd-AFP injected control mice. Averages ± standard deviations are shown. *p < 0.05.

Monomeric ATZ molecules bind together forming long, polymeric chains that reside in the ER of the cells in a conformation with a very long half-life. To determine the effect of HDAd-mediated gene transfer of TFEB on monomer and polymer ATZ pools, we analysed liver samples from HDAd-TFEB injected mice and corresponding controls using a previously published assay (An et al, 2005). First, ATZ polymers were isolated from monomers in liver lysates under non-denaturing conditions and then separated polymer and monomer fractions were denatured and compared by quantitative immunoblot. The denaturation step reduces polymers to monomers and the resulting bands can be compared at the same molecular weight (An et al, 2005). A significant decrease in both ATZ monomer and polymer was observed in livers of HDAd-TFEB injected mice compared to either saline or HDAd-AFP injected control mice (Fig 5).

**Figure 6:**
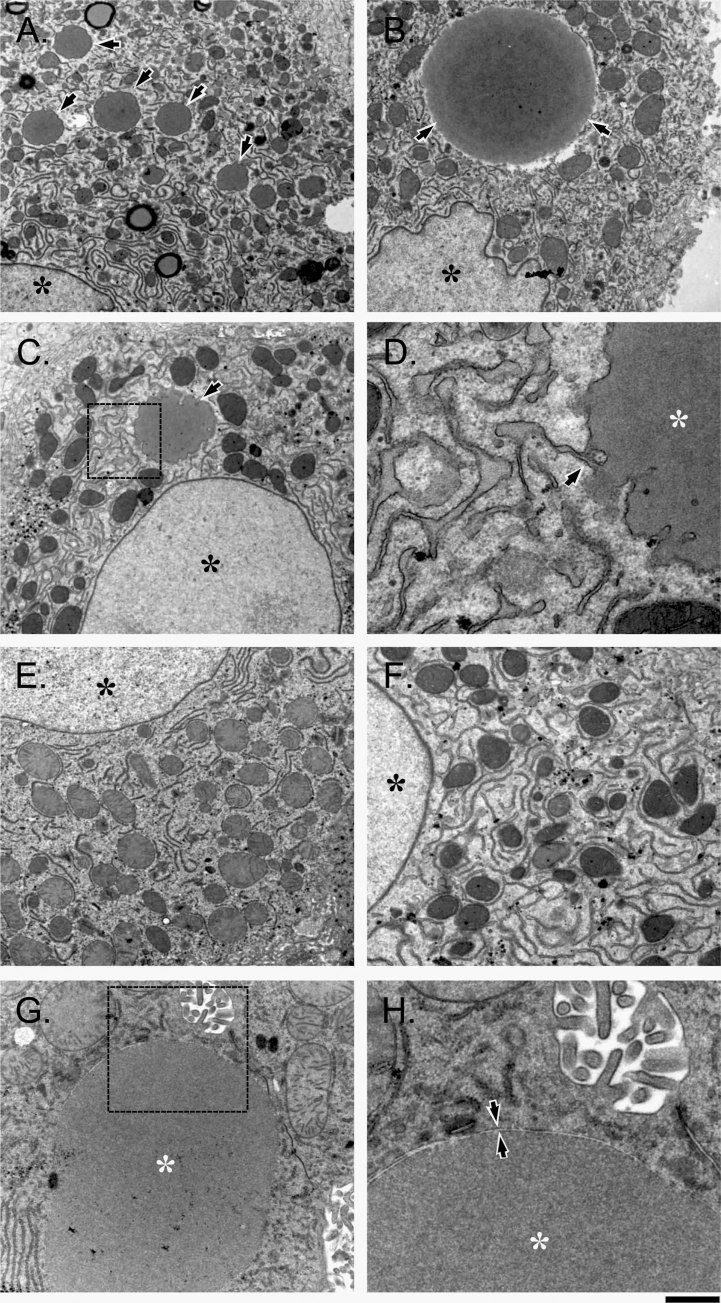
Hepatic TFEB gene transfer induced clearance of ATZ inclusions of PiZ mice Livers from either HDAd-AFP injected (**A**–**D**) or HDAd-TFEB injected (**E**–**H**) PiZ mice were prepared for EM as described in Material and Methods. **Scale bar**: 1.5 µm (**A**–**C**), 340 nm (**D**), 1.3 µm (**E,F**), 950 nm (**G**), 380 nm (**H**). **A–C.** Arrows show membrane bound inclusions in the hepatocyte cytoplasm, while asterisks in black indicate nuclei.**D.** This panel shows the area corresponding to the region outlined by dash box in (**C**). Arrow indicates the place where inclusion (asterisk in white) is connected with RER membranes.**E,F.** Hepatocytes in HDAd-TFEB injected animals do not exhibit inclusions in cytoplasm. Asterisks in black indicate nuclei.**G.** Example of hepatocyte that still contains inclusion (asterisk in white).**H.** The image shows the area corresponding to the region outlined by dash box in (**G**). Arrows indicate double membrane around inclusion (asterisk in white).

**Figure 7:**
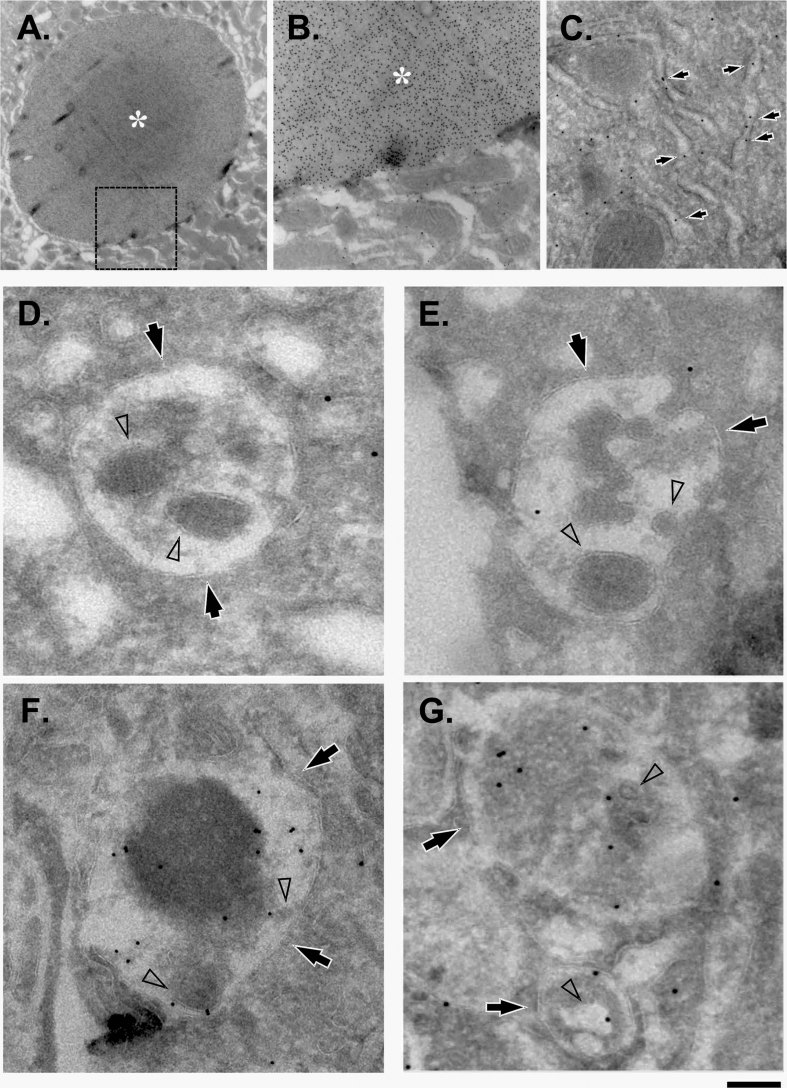
Increased ATZ in autophagolysosomes of HDAd-TFEB injected PiZ mice Livers from either HDAd-AFP injected (**A,B,D,E**) or HDAd-TFEB injected (**C,F,G**) mice were prepared for immuno-EM of ATZ as described in Materials and Methods Section. Scale bars: 1.8 µm (**A**), 520 nm (**B**), 320 nm (**C**), 125 nm (**F**), 110 nm (**D,E,G**). **A.** Region of the hepatocyte cytoplasm exhibits large membrane bound inclusion (asterisk) similar to that shown in Fig 6B.**B.** Higher magnification from the region outlined by dash box in **A** reveals dense gold ATZ labelling over inclusion body (asterisk).**C.** Arrows indicate diffuse ATZ signal along the RER membranes of hepatocyte in HDAd-TFEB-treated mice.**D,E.** Lysosomes (arrows) and their intraluminal vesicles (arrowheads) show little or no ATZ in control saline-injected animals.**F,G.** Liver cells from HDAd-TFEB injected mice exhibit ATZ-corresponding gold particles within the lysosome-like structures (arrows) where ATZ signal is frequently associated with intraluminal vesicles (arrowheads).

Electron microscopy (EM) analysis of thin sections from livers of animals injected with saline or control HDAd-AFP vector revealed numerous membrane bound inclusions in hepatocyte cytoplasm (Fig 6A–D). These inclusions ranged from smaller (0.3–1 µm; see Fig 6A) to larger sizes which were comparable to nuclei (up to 10 µm; see Fig 6B), and exhibited membrane continuity with cisternae of rough endoplasmic reticulum (RER) decorated by ribosomes (Fig 6C and D). These features suggested that such structures are the sites of newly synthesized ATZ which accumulates and aggregates in the RER. Immunolabelling of ATZ in thin cryosections indicated a strong concentration of ATZ in the inclusions of control HDAd-AFP injected PiZ mice (Fig 7A and B). In contrast, most hepatocytes in HDAd-TFEB injected animals lack the inclusions observed in control PiZ mice (Fig 6E and F), with exception of few cells that still contained large ATZ aggregates (Fig 6G). Notably, these remaining aggregates were frequently surrounded by double membrane (Fig 6G and H), that indicates their transformation into an autophagic vacuole.

**Figure 8:**
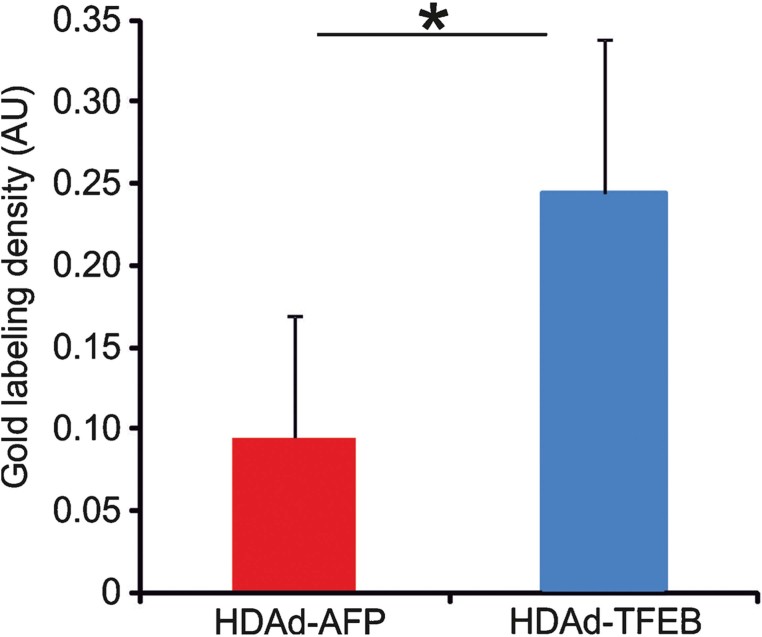
ATZ content was increased in autophagolysosomes of HDAd-TFEB injected PiZ mice . Immuno-gold ATZ labelling densities (*n* = 20 cells) in lysosome-like structures were quantified as described in Materials and Methods section and expressed in arbitrary units (AU). Averages ± standard deviations are shown. *p < 0.05.

Immuno-EM revealed ATZ to be diffusely distributed along the RER profiles in the hepatocytes of HDAd-TFEB injected mice (Fig 7C). In control HDAd-AFP injected mice, several multi-vesicular body (MVB)-like structures, that correspond to ‘lysosomes’ or ‘autolysosomes’ based on their ultrastructural features (Saftig & Klumperman, 2009), exhibited little or no ATZ (Fig 7D and E). In sharp contrast to control mice, significant amounts of ATZ protein were detected within MVB-like structures in HDAd-TFEB injected mice (Fig 7F and G). The elevated ATZ signal in MVB-like structures indicates the activation of ATZ degradation by the lysosomal pathway upon TFEB gene transfer. Indeed, gold particles in lysosome-like organelles were frequently associated with intraluminal vesicles that are actively involved in lysosome degradation (Saftig & Klumperman, 2009), as shown by morphometric quantitative analysis (Fig 8). Taken together these data, showed that TFEB hepatic expression enhances degradation of insoluble ATZ in autolysosomes.

Previous studies have shown mitochondrial autophagy and injury in the liver of PiZ mice and affected humans. However, it remains unclear whether ATZ retention in the ER is itself responsible for mitochondrial dysfunction or the autophagic response results in non-specific removal of normally functioning mitochondria in ATZ injured livers (Teckman et al, 2004). The predominant ultrastructural feature of mitochondria in PiZ livers is the degeneration, which did not appear to occur within autophagolysosomes. Affected mitochondria appear as multilamellar structures within their limiting membrane and condensation of matrix and cristae (Teckman et al, 2004). Therefore, we analyzed livers of HDAd-TFEB and control injected mice for evidence of mitochondrial damage at the ultrastructural level. Mitochondria of PiZ mouse hepatocytes exhibited frequently swollen cristae (Supporting Information Fig S6A, arrows) that suggest mitochondrial dysfunction. In contrast, the mitochondrial structure of HDAd-TFEB injected mice was normal with thinner cristae (Supporting Information Fig S6B, arrows). Moreover, HDAd-TFEB injected mice did not show a reduction in the number of mitochondria in livers (Supporting Information Fig S6C), indicating that their consumption through mitophagy was not accelerated. In addition, increased levels of mitochondrial citrate synthase and CoxIV proteins were detected in HDAd-TFEB injected mice compared to HDAd-AFP or saline injected mice (Supporting Information Fig S6D and E). Taken together, these data show that enhancement of autophagy mediated by TFEB gene transfer does not result in mitochondrial depletion but conversely, it may have a positive effect on mitochondria. Mitochondria are physically in close vicinity to the cisternae of the ER with which they interact (Arnaudeau et al, 2001; Perkins et al, 1997) and based on previous studies (Teckman et al, 2004) and our results, mitochondrial dysfunction in AAT deficiency appears to be likely secondary to ATZ retention in the ER rather than due to abnormal non-specific increase in mitochondrial autophagy. Indeed this observation is consistent with phenotypic improvements by autophagy enhancers, such as rapamycin or carbamazepine, reported in previous studies on AAT deficiency (Hidvegi et al, 2010; Kaushal et al, 2010) and neurodegenerative disorders (Khandelwal et al, 2011; Liu & Lu, 2010; Thomas et al, 2011).

**Figure 9:**
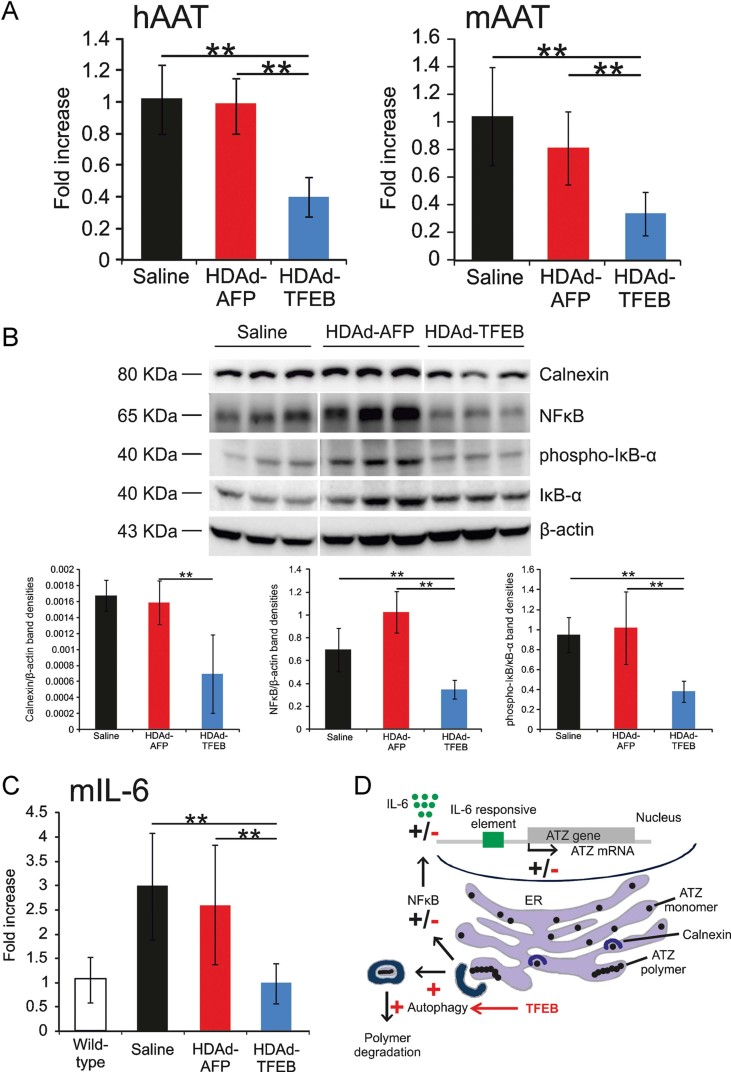
TFEB gene transfer interrupted ATZ pathogenic vicious cycle **A.** ATZ and endogenous mouse AAT (mAAT) mRNA levels, measured by real time PCR and expressed as fold increase over saline-treated mice were both reduced in HDAd-TFEB injected PiZ mice compared to either saline or HDAd-AFP injected control PiZ mice.**B,C.** TFEB gene transfer resulted in reduction of calnexin, NFκB, phospho-IκB-α proteins (**B**) and IL-6 mRNA (**C**) in the livers of PiZ mice. Real time PCR were performed on mRNA from at least *n* = 5 mice and are expressed as fold increase over wild-type mice. For Western blots representative bands from three mice for each treatment group are shown. The graph shows densitometric quantification of *n* = 5 mice from each treatment group. Averages ± standard deviations are shown.**D.** ATZ accumulation in the ER activates NFκB pathway (Hidvegi et al, 2005) and such activation aggravates the burden of intracellular ATZ. NFκB-dependent increase of IL-6 enhances ATZ expression through binding to the IL-6 responsive element of the AAT promoter (Alam et al, 2012; Morgan et al, 1997). The activating (+) or inhibiting effects (−) induced by TFEB on the molecules involved in this positive feedback mechanism are shown in red. The molecular events occurring in the absence of TFEB gene transfer are shown in black. *p < 0.05; **p < 0.01.

While reduction of accumulated ATZ polymer is dependent upon increased autophagolysosome degradation, a different mechanism has to be involved in reduction of ATZ monomer detected by monomer analysis (Fig 5). We found that ATZ mRNA levels were reduced in livers of HDAd-TFEB injected mice compared to either saline or HDAd-AFP injected control mice (Fig 9A). Because the cis-acting elements regulating ATZ expression are retained in the transgenic PiZ mice (Carlson et al, 1988), we next determined whether the expression of endogenous mouse AAT (mAAT) was also affected by TFEB gene transfer. Indeed, we found that mAAT mRNA was reduced in HDAd-TFEB injected mice (Fig 9A), thus suggesting that ATZ monomer reduction occurred through a transcriptional effect.

Previous studies have shown that ATZ accumulation induces ER overload response (EOR) that activates the NFκB pathway (Hidvegi et al, 2005; Lawless et al, 2004; Ron, 2002) through IκB-α phosphorylation (Lawless et al, 2004). NFκB activation further aggravates ATZ intracellular accumulation because it increases IL-6 transcription (Lawless et al, 2004), which in turn enhances ATZ transcription through binding to an IL-6 responsive element of the AAT promoter (Fig 9D; Alam et al, 2012; Morgan et al, 1997). TFEB gene transfer interrupts this deleterious positive feedback loop and results in reduction of calnexin, a molecular chaperone of AAT and a sensor of EOR (Alam et al, 2012; Lawless et al, 2004; Fig 9B), decreased NFκB synthesis and activation (Fig 9B), and normalizes mIL-6 mRNA levels (Fig 9C). NFκB is sequestered in the cytoplasm by the IκB inhibitory proteins (Brockman et al, 1995; Whiteside et al, 1997) and thus reduction of phosphorylated-IκB-α reflects a reduction in the activation of NFκB (Fig 9B). In summary, TFEB-mediated augmentation of ATZ polymer degradation interrupts the vicious cycle that increases the burden of intracellular ATZ resulting in ER stress, NFκB activation and IL-6 dependent activation of ATZ transcription (Fig 9D). In contrast to PiZ mice, TFEB gene transfer in C56BL/6 wild-type mice did not result neither in changes of NFκB protein levels (Supporting Information Fig S7A) nor in changes of IL-6 mRNA (Supporting Information Fig S7B), thus suggesting that reduced NFκB activation in PiZ mice is not a direct effect of TFEB gene transfer.

**Figure 10:**
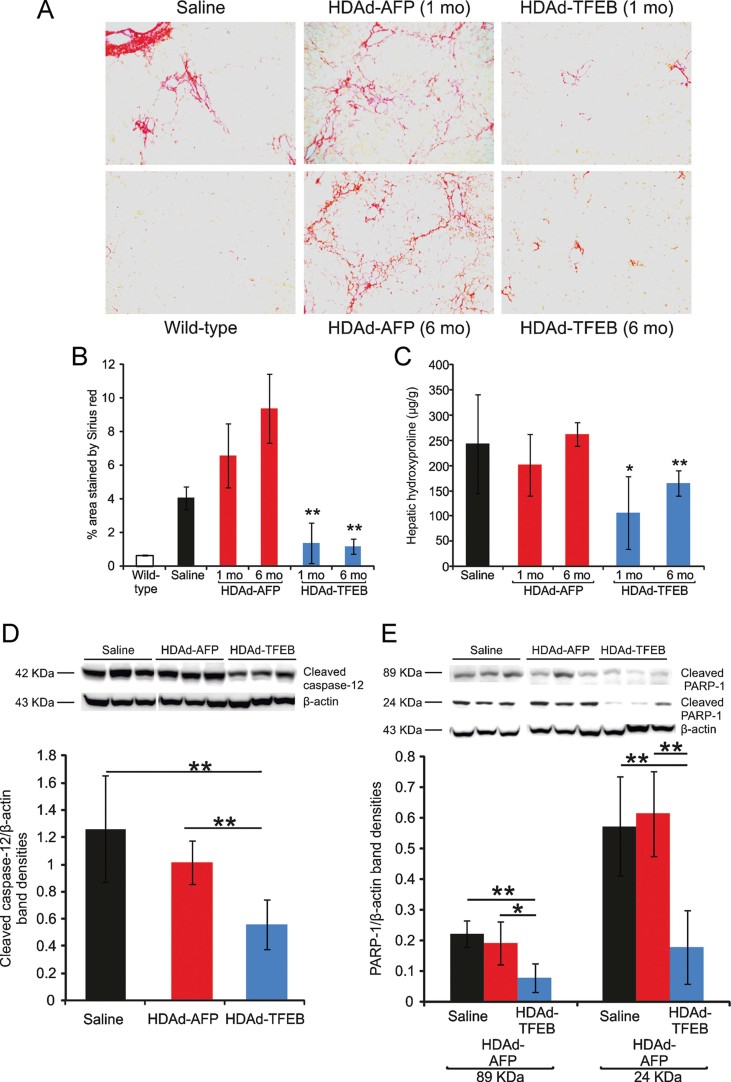
Correction of liver disease following TFEB gene transfer **A.** Sirius red staining in livers from PiZ mice at 1 month (1 mo) and 6 months (6 mo) after the injection of HDAd-TFEB or HDAd-AFP vector. Sirius red staining from a wild-type control is also shown (magnification 20×). Representative images are shown from experimental groups including at least *n* = 5 mice.**B,C.** Morphometric analysis of Sirius red staining (**B**) and hepatic hydroxyproline content (**C**) showed a reduction of fibrosis in PiZ mice injected with HDAd-TFEB compared to saline or HDAd-AFP injection (at least *n* = 5 per group) at both 1 month (1 mo) and 6 months (6 mo) post-injection.**D.** Western blot analysis showed that PiZ mice injected with HDAd-TFEB have a significant reduction of cleaved caspase-12 compared to saline or HDAd-AFP injected mice (representative bands from three independent animals are shown). Band densities of cleaved 42 kDa caspase-12 band normalized for β-actin levels showed a statistical significant reduction in HDAd-TFEB injected mice (*n* = 5 per group).**E.** Western blot analysis showed that PiZ mice injected with HDAd-TFEB have a significant reduction of cleaved PARP-1 compared to saline or HDAd-AFP injected mice (representative bands from three independent animals are shown). Densities of cleaved 89 and 24 kDa PARP-1 bands normalized for β-actin levels showed a statistical significant reduction in HDAd-TFEB injected mice (*n* = 5 per group). Averages ± standard deviations are shown. *p < 0.05, **p < 0.01.

Hepatic fibrosis is a key feature of the hepatic disease in AAT deficiency and is secondary to hepatocyte apoptosis. Therefore, we next investigated whether TFEB gene transfer reduces ATZ-induced liver fibrosis. Collagen deposition was determined by Sirius red staining and by measurement of hepatic hydroxyproline content. HDAd-TFEB injection resulted in a long-term reduction of Sirius red staining (Fig 10A and B) and hydroxyproline content in the livers of HDAd-TFEB injected mice compared to saline and HDAd-AFP injected animals (Fig 10C).

Caspase-12 is involved in ER stress-induced apoptosis in both ATZ expressing cells and livers (Hidvegi et al, 2005). Therefore, we investigated whether the reduction of ATZ load in HDAd-TFEB injected mice resulted in decreased activation of caspase-12. Western blot analysis showed that the ∼42 kDa cleavage product that corresponds to activated caspase-12 was significantly diminished in HDAd-TFEB injected mouse livers compared to control livers (Fig 10D). We also observed a reduction in caspase-cleaved 89 and 24 kDa fragments of poly(ADP-ribose) polymerase-1 (PARP-1), which are generated during the execution of apoptotic program (Fig 10E). In summary, TFEB hepatic gene transfer decreased detrimental activation of liver apoptosis and fibrosis which underlines the pathogenesis of neonatal hepatitis, cirrhosis and hepatocellular carcinoma in AAT deficiency (Rudnick & Perlmutter, 2005).

## DISCUSSION

Gene therapy strategies for liver disease of AAT deficiency have been investigated to avoid liver transplantation and have been designed with the goal of downregulating endogenous hepatocyte ATZ levels (McLean et al, 2009). Previous attempts to correct the liver phenotype of AAT deficiency have been focused on short hairpin RNA (shRNA) to silence ATZ (Cruz et al, 2007; Duan et al, 2004). However, shRNA delivered by gene therapy vectors has raised concerns because severe toxicity and lethality have been reported in mice (Grimm et al, 2006). shRNA-mediated saturation of the exportin-5 pathway, which shuttles cellular micro-RNA (miRNA) from nucleus to cytoplasm, has been proposed as responsible for this severe toxic response (Grimm et al, 2006). To overcome this problem, strategies based on viral vector-mediated transfer of miRNA sequences targeting the AAT gene have been developed (Mueller et al, 2012).

In the present study, we have investigated a novel strategy to correct the hepatic disease of AAT deficiency, based on clearance and prevention of ATZ accumulation by TFEB gene transfer. The current view is that ATZ is degraded by both proteasomal and autophagic pathway. The proteasome is responsible for degrading the soluble form of ATZ by means of ER-associated degradation while autophagy is involved in disposal of insoluble ATZ polymers and aggregates (Perlmutter, 2009). Autophagy has been previously shown to be involved in ATZ degradation (Hidvegi et al, 2010; Kamimoto et al, 2006; Teckman and Perlmutter, 2000; Teckman et al, 2002).

To investigate whether TFEB increases ATZ degradation via the autophagic pathway, we first carried out pulse-chase analysis in wild-type MEF transiently co-transfected with plasmids expressing TFEB and ATZ. The results of these studies showed that TFEB increases the clearance of intracellular ATZ and reduces ATZ secretion in media (Fig 1A and C). To provide genetic evidence for autophagy-mediated disposal of ATZ, we used cells deleted for *Atg7* gene that is necessary for initiation of autophagy (Komatsu et al, 2005). In the absence of autophagy, degradation of ATZ was abrogated (Fig 1B and D).

Next, we showed that hepatic TFEB gene transfer resulted in long-term reduction of polymeric ATZ accumulation upon increased autophagy flux and lysosomal biogenesis in the liver, as indicated by increased LAMP-1 and LC3, reduced SQSTM1/p62, decreased autophagolysosome number (Fig 4), and increased ATZ signals within autolysosomes (Figs 7 and 8). Although slightly reduced, hepatic TFEB expression was sustained for up to 6 months post-injection (Fig 2D). Adenoviral vector genomes do not integrate into the genome of the transduced cells and therefore, vector genomes are lost in actively dividing cells (Stephen et al, 2010). The slight reduction in hepatic TFEB expression is likely dependent on hepatocyte turn-over. Nevertheless, clear reductions of PAS staining and ATZ levels were still detected at least up to 6 months post-injection (Fig 2A). Should TFEB expression fade over time, to maintain phenotypic correction it would be possible to re-administer a vector with a different serotype to overcome the neutralizing anti-Ad antibody elicited with the first administration (Kim et al, 2001; Morral et al, 1999).

The results of the present study indicate that HDAd vector-mediated TFEB hepatic expression resulted in reduction of ATZ aggregates that were already present, prevention of further formation of ATZ globules, and thus it is effective at reducing liver injury, as shown by decreased hepatic apoptosis and fibrosis (Fig 10). At the time of injection (3 months of age) PiZ mice showed in fact significant amount of PAS staining (Supporting Information Fig S3B) which was cleared by TFEB-induced autophagy. Liver injury in patients with AAT deficiency is a direct consequence of hepatic accumulation of polymerized ATZ and therefore, TFEB gene transfer has tremendous potential for the treatment of liver disease in patients with AAT deficiency. In contrast to previous strategies aimed at downregulating ATZ expression and thus effective only to prevent further polymer accumulation, TFEB gene transfer resulted in both clearance of ATZ polymer and reduction of ATZ monomer. ATZ monomer reduction occurred as a result of TFEB-mediated clearance of ATZ polymers that reduced NFκB activation and IL-6 signaling, thus interrupting a positive feedback loop which aggravates liver injury by increasing ATZ transcription (Fig 9).

Previous work demonstrated that overexpression of TFEB promotes reduction of glycosaminoglycans in a mouse model of lysosomal storage disorder due to deficiency of lysosomal enzymes (Medina et al, 2011). In the present study, we show that TFEB gene transfer results in clearance of mutant, toxic protein which accumulates in a cell compartment that is not the lysosome. Therefore, our data indicate that TFEB gene transfer may be effective for treatment of a wide spectrum of human disorders due to intracellular accumulation of toxic proteins, including neurodegenerative disorders.

Although the results of the present study were obtained with the HDAd vector, similar outcomes are expected with TFEB gene transfer by other gene therapy vectors, such as recombinant adeno-associated viral (AAV) vectors that has recently generated encouraging results in humans (Nathwani et al, 2011). Ultimately, the choice of the vector for clinical liver gene therapy will be dictated by a careful evaluation of efficacy and safety profile between the available vectors.

Although TFEB gene transfer strategy proved effective to correct the liver disease, this approach would not affect the loss-of-function pulmonary phenotype of AAT deficiency. Nevertheless, lung disease due to AAT deficiency can be prevented by protein augmentation therapy (American Thoracic/European Respiratory Society, 2003; Wewers et al, 1987) or replenishment of the deficient gene product via AAV-mediated muscle-directed gene therapy (Brantly et al, 2009; Flotte et al, 2004).

Up to 6 months after vector administration, the HDAd-TFEB-injected mice appeared in general good health and were undistinguishable from the control groups. Long-term assessment of toxicity in these animals did not show evidence of hepatic toxicity (Supporting Information Fig S4 and S5). However, further studies in large animals are needed to investigate safety of long-term TFEB expression. Nevertheless, the results of the present study illustrate the great potential of TFEB gene transfer, or possibly of pharmacological approaches resulting in TFEB activation, for therapy of liver disease caused by hepatotoxic ATZ. TFEB co-localizes with master growth regulator mTOR complex 1 (mTORC1) on the lysosomal membrane and in the presence of nutrients TFEB phosphorylation by mTORC1 inhibits TFEB activity (Settembre et al, 2012). Conversely, pharmacological inhibition of mTORC1, as well as starvation and lysosomal disruption, activates TFEB by promoting its nuclear translocation. Small molecules inducing TFEB nuclear translocation and activity have been identified and could be used for therapeutic applications (Settembre et al, 2012). Therefore, besides gene transfer, pharmacological induction of TFEB or TFEB target gene activation can be exploited to promote clearance of ATZ.

Genetic or environmental modifiers predispose a subgroup of homozygotes for the classical form of AAT deficiency to develop liver disease and/or protect the remainder population from hepatic disease (Piitulainen et al, 2005). Moreover, ATZ protein appears to also act as a modifier gene that exacerbates other forms of liver disease (Campbell et al, 2007; Graziadei et al, 1998). The results of our study could suggest that genetically determined differences in the level of activity of the TFEB-autophagy-lysosome axis may play a role in favouring or protecting from the development of liver disease in AAT deficiency.

In conclusion, our study shows the efficacy of TFEB gene transfer for therapy of liver disease of AAT deficiency by ATZ disposal through the autophagolysosome system. TFEB gene transfer might provide an innovative therapeutic strategy for treatment of hepatic damage caused by AAT deficiency, which is a common cause of liver injury.

## MATERIALS AND METHODS

### Cell culture studies

The human AAT cDNA was inserted into a pcDNA3.1 plasmid (Invitrogen) and the ATZ was generated by site-directed mutagenesis (Stratagene). Wild-type and Atg7^−/−^ MEF were cultured in DMEM with 10% foetal bovine serum (FBS), 5% penicillin/streptomycin and 2 mM l-glutamine. Wild-type and Atg7^−/−^ MEF were transiently co-transfected with pcDNA3.1-ATZ and pCMV-GFP (as a control) or with pcDNA3.1-ATZ and p-CMV-TFEB-HA plasmids using Lipofectamine 2000 (Invitrogen). Twenty-four hours after transfection, cells were pulse labelled with 150 µCi/ml of Easy Tag Express Protein Labelling Mix (Perkin Elmer) in pulse medium for 90 min at 37°C. Cells were then rinsed with DMEM 5% FBS with Met and Cys (chase medium) and chased for different time points. Cells were lysed in lysis buffer (50 mM Tris–HCl pH 7.4, 200 mM NaCl, 1% Triton X-100, 1 mM EDTA, 50 mM HEPES, 1× protease inhibitor) for 1 h at 4°C. Cell lysates were rotated overnight at 4°C with the rabbit anti-human AAT (Dako). Following the addition of protein A sepharose (Sigma), samples were incubated for 2 h at 4°C. After four washes with lysis buffer, samples were resuspended in Laemmli Buffer 2× (24 mM Tris–HCl pH 6.8, 0.8% SDS, 4% glycerol, 2.5% β-mercaptoethanol, 0.004% bromophenol blue), boiled for 5 min and cell extracts were loaded onto a 10% SDS–PAGE gel.

Wild-type MEF were transiently co-transfected with pcDNA3.1-ATZ and pCMV-GFP or with pcDNA3.1-ATZ and pCMV-TFEB-HA plasmids using Lipofectamine 2000 (Invitrogen). Twenty-four hours after transfection, cells were incubated with 30 µM MG132 (Selleckchem) or DMSO for 6 h, washed once with cold phosphate buffered saline (PBS), and scraped with RIPA buffer (50 mM Tris–HCl pH 7.4, 150 mM NaCl, 1% Triton X-100, 1 mM EDTA pH 8.0, 0.1% SDS) containing complete protease inhibitor cocktail (Sigma). Samples were incubated for 20 min at 4°C, centrifuged at 13,200 rpm for 10 min and cell lysates were used for Western blot. After transfer to polyvinylidene difluoride (PVDF) membrane, blots were blocked with TBS-Tween 20 containing 5% non-fat milk for 1 h at room temperature followed by incubation with rabbit-anti-human AAT (Dako) overnight at 4°C. Donkey anti-rabbit IgG conjugated with horseradish peroxidase (HRP; GE Healthcare) and ECL (Pierce) were used for detection of ATZ. Equal gel loading was confirmed with immunoblot for β-actin (Novus Biologicals).

TFEB-3xFLAG HeLa stable cell lines (HeLa-CF7; Sardiello et al, 2009) and HeLa untransfected cells were cultured in DMEM with 10% foetal bovine serum (FBS) and 5% penicillin/streptomycin and transiently transfected with pcDNA3.1-ATZ using Lipofectamine 2000 (Invitrogen). Twenty-four hours after transfection, cells were washed once with cold PBS and scraped with RIPA buffer containing protease inhibitor cocktail (Sigma). Samples were incubated for 20 min at 4°C and centrifuged at 13,200 rpm for 10 min. The pellet was discarded and cell lysates were used for Western blot analysis.

### HDAd vectors

HDAd-TFEB and HDAd-AFP vectors both bear a PEPCK-WL expression cassette (Brunetti-Pierri et al, 2006; Palmer & Ng, 2003), as shown in Supporting Information Fig S1, driving the expression of human TFEB or baboon AFP, respectively. HDAd was produced in 116 cells with the helper virus AdNG163 as described elsewhere (Palmer & Ng, 2003). Helper virus contamination levels were determined as described elsewhere and were found to be <0.05%. DNA analyses of HDAd genomic structure was confirmed as described elsewhere (Palmer & Ng, 2003).

### Mice and injections

Animal procedures were performed in accordance to the regulations of the Italian Ministry of Health. PiZ transgenic mice (Carlson et al, 1988) were maintained on a C57/BL6 background. Injections of HDAd-TFEB, HDAd-AFP or saline were performed in a volume of 200 µl in the retrorbital plexus of 3-month-old male PiZ mice. Mice were sacrificed at 4 weeks or 6 months post-injection and liver samples were harvested for analyses. For assessment of hepatic NFκB, 3-month-old C57/BL6 wild-type mice (Charles-River) were injected with either HDAd-TFEB or HDAd-AFP vector and sacrificed at 4 weeks post-injection. Blood samples were collected by retrorbital bleeding.

### Analyses of serum and liver extracts

Liver protein extracts and serum samples were analysed by ELISA for ATZ detection. To detect human ATZ by ELISA, multi-well plates (Nunc Maxisorp) were coated with cappel goat anti-human AAT (MP Biomedicals), and then blocked in 0.1% PBS Tween 20 containing 5% non-fat milk. Serial dilutions of purified human AAT were loaded to build a standard curve. Rabbit-anti-human AAT (Dako; 1:4000) was used as capturing antibody and goat anti-rabbit IgG-HRP (Dako; 1:2000) as secondary antibody. Serum was analysed for ALT and AST according to manufacturer's instructions (Gentaur).

### Liver stainings

PAS staining was performed on 10-µm thick paraffin sections of livers. Liver specimens were fixed in 4% PFA for 12 h and stored in 70% Ethanol, embedded into paraffin blocks and cut into 10 µm sections. The sections were rehydrated and treated with amylase solution 0.5% (α-amylase type VI-B, Sigma) for 20 min and then stained with PAS reagent according to manufacturer's instructions (Bio-Optica). For ATZ immunofluorescence, 6-µm sections were rehydrated, blocked, incubated overnight at 4°C with rabbit anti-human AAT (Dako) and then with donkey anti-rabbit 488 (AlexaFluor) for 1 h at room temperature.

For co-staining LAMP-1 and ATZ, 6-µm thick paraffin sections of livers were de-waxed by standard techniques, hydrated in PBS pH 7.4 and permeabilized with PBS, 0.2% Triton. Heat Induced Epitope Retrieval using citrate buffer method (pH 6.0) for LAMP-1 and Protease-induced Epitope Retrieval by Proteinase K for ATZ were performed to retrieve the antigen sites. The sections were then covered for 30 min with 75 mM NH_4_Cl/PBS to reduce quenching and incubated for 1 h at room temperature with blocking solution (3% BSA, 5% donkey serum, 20 mM MgCl_2_, 0.3% Tween 20 in PBS pH 7.4). The primary antibodies used were: rat monoclonal LAMP-1 (1D4B; Santa Cruz Biotechnology) and polyclonal rabbit anti-human AAT (Dako). The incubation for LAMP-1 was carried out overnight at 4°C whereas that for AAT was done for 1 h at room temperature. The secondary antibodies made in donkey were: AlexaFluor-594 anti-rat (Invitrogen) for LAMP-1 and AlexaFluor-488 (Invitrogen) for AAT. Nuclei were counterstained with DAPI (Invitrogen). Finally, the stained liver sections were mounted in mowiol, cover-slipped and examined under a Zeiss LSM 710 confocal laser-scanning microscope.

**The paper explained**

PROBLEM:

Alpha-1-anti-trypsin deficiency is the most common genetic cause of liver disease in children and liver transplantation is the only available treatment.

RESULTS:

Liver-directed gene transfer of TFEB, a master gene that regulates lysosomal function and autophagy, in the mouse model of alpha-1-anti-trypsin deficiency resulted in reduction of mutant, hepatotoxic alpha-1-anti-trypsin and rescue of liver apoptosis and fibrosis, which are key features of alpha-1-anti-trypsin deficiency. Correction of the hepatic phenotype was dependent upon increased ATZ polymer degradation by enhanced autophagy and reduced ATZ monomer mediated by a reduction of hepatic NFκB activation and IL-6 that drives ATZ gene expression.

IMPACT:

TFEB gene transfer is a novel strategy for treatment of liver disease of alpha-1-anti-trypsin deficiency. This study may pave the way towards applications of TFEB gene transfer for treatment of human disorders due to accumulation of toxic proteins.

Sirius red staining was performed on 10-µm liver sections which were rehydrated and stained for 1 h in picro-sirius red solution (0.1% Sirius red in saturated aqueous solution of picric acid). After two changes of acidified water (5 ml acetic acid glacial in 1 L of water), the sections were dehydrate in three changes of 100% ethanol, cleared in xylene and mounted in a resinous medium. To quantify Sirius red staining, Image J Software (NIH) was used to calculate the percent area staining positively in five random low power views, as previously described (Mencin et al, 2007).

### Electron microscopy (EM) studies

For routine EM analysis small pieces of liver were excised from PiZ mice injected with either saline or control HDAd-AFP vector or HDAd-TFEB and fixed in 1% glutaraldehyde in 0.2 M HEPES buffer. Then, small blocks of liver tissues were post-fixed in uranyl acetate and in OsO_4_. After dehydration through a graded series of ethanol, tissue samples were cleared in propylene oxide, embedded in Epoxy resin (Epon 812) and polymerized at 60°C for 72 h. From each sample, thin sections were cut with a Leica EM UC6 ultramicrotome. For immuno-EM analysis of ATZ distribution in hepatocytes, small pieces of liver tissues were fixed in a mixture of 4% paraformaldehyde and 0.4% glutaraldehyde in 0.2 M PHEM buffer, infused with 2.3 M sucrose, frozen in liquid nitrogen and sectioned in Leica EM FC7 cryoultratome. Cryosections were incubated with antibodies against ATZ and then with protein A conjugated with 10-nm gold particles. Both cryo and Epon-812 plastic sections were further investigated using a FEI Tecnai-12 (FEI, Einhoven, The Netherlands) electron microscope equipped with an Veletta CCD camera for digital image acquisition. Quantification of ATZ gold labelling densities over the lysosome-like organelle in hepatocytes was performed using iTEM software (Olympus SYS, Germany) according to previously described method (Mironov et al, 2001). Briefly, morphometric grid with 50-nm mesh was placed over profiles of lysosome-like structures. ‘Touch count’ module of iTEM software was used to quantify (i) number of gold particles and (ii) number of grid nodes inside the lysosome profile. Gold density was expressed in arbitrary units (gold particles per node). The organelle was defined as ‘lysosome’ on the basis of the round/oval shape and presence of intraluminal vesicles as well as disorganized electron-dense and membrane material in the lumen (Saftig & Klumperman, 2009).

Autophagosomes were identified in plastic sections on the basis of their ultrastructural features, mainly double membranes that surround their interior. The number of autophagosomes and mitochondria per cells in livers of control and HDAd-TFEB injected mice was quantified using iTEM software.

### Western blots

Liver specimens were homogenized in RIPA buffer and complete protease inhibitor cocktail (Sigma). Samples were incubated for 20 min at 4°C and centrifuged at 13,200 rpm for 10 min. The pellet was discarded and cell lysates were used for Western blot analysis. Ten micrograms of liver protein were loaded for each specimen into a 12% SDS–PAGE. After transfer to PVDF membrane, blots were blocked with TBS-Tween-20 containing 5% non-fat milk for 1 h at room temperature followed by incubation with primary antibody overnight at 4°C. The primary antibodies used were: rabbit-anti-human AAT (Dako), mouse-anti-p62 (BD Transduction Laboratories), rabbit anti-LC3 (Novus Biologicals), rabbit-anti-calnexin (Enzo, Life Science), rabbit-anti-NFκB (Cell Signaling Technology), mouse-anti-phospho-Iκbα (Cell Signaling Technology), mouse anti-Iκbα (Cell Signaling Technology), rat anti-caspase-12 (Sigma), rabbit anti-PARP-1 (Alexis Biochemical), rabbit anti-citrate synthase (Abcam), rabbit anti-CoxIV (Cell Signaling Technology). Equal loading of protein was determined by Western blot using a mouse anti-β-actin antibody (Novus Biologicals). Band densitometry was performed using Quantity One Software (BioRad Laboratories). For caspase-12 and PARP Western blotting, 20 µg of total protein were loaded on 10% SDS–PAGE and after transfer to PVDF membrane, the blots were blocked in TBS-Tween20 containing 5% non-fat milk or 1% BSA for 1 h at room temperature followed by overnight 4°C incubation with rat-anti-caspase-12 (Sigma) or rabbit-anti-PARP-1 (Alexis Biochemical). Goat anti-rat IgG-HRP (GE Healthcare) or anti-rabbit IgG-HRP (GE Healthcare) were used for detection.

### Other liver assays

Momomer–polymer analysis was performed according to previous method (An et al, 2005). Hydroxyproline content was measured by a spectrophotometric assay as an assessment of liver collagen content as previously described (Reddy & Enwemeka, 1996) and expressed as microgram of hydroxyproline per gram of liver.

### Real time PCR

Total RNA was extracted from liver tissue in TRIzol reagent (Invitrogen) using RNeasy kit (Qiagen). RNA was reverse transcribed using a first-strand complementary deoxyribonucleic acid kit with random primers according to the manufacturer's protocol (Applied Biosystems). The RT-PCR reactions were performed using the Roche Light Cycler 480 system (Roche). The PCR reaction was performed using the SYBR Green Master Mix (Roche). The PCR conditions for all of the genes were as follows: pre-heating, 5 min at 95°C; cycling, 40 cycles of 15 s at 95°C, 15 s at 60°C and 25 s at 72°C. Results were expressed in terms of cycle threshold (*C*_t_). The *C*_t_ values were averaged for each duplicate. The β_2_-microglobulin gene was used as endogenous control (reference marker). Differences between the mean *C*_t_ values of the tested genes and those of the reference gene were calculated as 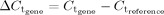. Relative fold increase in expression levels were determined as 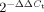. The analyzed genes were: mFIX (For 5′-GGCTCTCATCACCATCTTCC-3′, Rev 5′-TGGACGGGTAAGAATTTTGG-3′), mTFRC (For 5′-GGATCAAGCCAGATCAGCAT-3′, Rev 5′-CAGCCAGTTTCATCTCCACA-3′), mOTC (For 5′-GGATAGGGGATGGGAACAAT-3′, Rev 5′-CTGGCTCATAACCCTTTGGA-3′), mouse AAT (For 5′-GGAGCAAACTCTCAGCAAGG-3′, Rev 5′-ATGGACAGTCTGGGGAAGTG-3′), human ATZ (For 5′-CCAAGGCCGTGCATAAGG-3′, Rev 5′-GGCCCCAGCAGCTTCAGT-3′), mIL-6 (For 5′-CCGGAGAGGAGACTTCACAG-3′, Rev 5′-CAGAATTGCCATTGCACAAC-3′).

Primers to recognize TFEB were designed to amplify both human and mouse TFEB (For 5′-GGTGCAGTCCTACCTGGAGA-3′, Rev 5′-GTGGGCAGCAAACTTGTTCC-3′).

### Statistical analyses

Data are expressed as mean values ± standard deviation. Statistical significance was computed using the Student's 2 tail *t*-test. A *p*-value < 0.05 was considered statistically significant.

## For more information

OMMBID:

http://www.ommbid.com/OMMBID/the_online_metabolic_and_molecular_bases_of_inherited_disease/b/abstract/part26/ch219

Alpha1 Foundation:

http://www.alpha-1foundation.org/

## Supplementary material

As a service to our authors and readers, this journal provides supporting information supplied by the authors. Such materials are peer reviewed and may be re-organized for online delivery, but are not copy-edited or typeset. Technical support issues arising from supporting information (other than missing files) should be addressed to the authors.
