# Supplementary material for: Gene transfer of master autophagy regulator TFEB results in clearance of toxic protein and correction of hepatic disease in alpha-1-anti-trypsin deficiency
Source: EMBO Mol Med. 2013 Feb 4;5(3):397–412. doi: 10.1002/emmm.201202046 (PMC3598080; doi:10.1002/emmm.201202046)
Supplement: Supplementary file 1 [file emmm0005-0397-SD1.pdf]

# Gene transfer of master autophagy regulator TFEB results in clearance of toxic protein and correction of hepatic disease in alpha-1-antitrypsin deficiency

Nunzia Pastore, Keith Blomenkamp, Fabio Annunziata, Pasquale Piccolo, Pratibha Mithbaokar, Rosa Maria Sepe, Francesco Vetrini, Donna Palmer, Philip Ng, Elena Polishchuk, Simona Iacobacci, Roman Polishchuk, Jeffrey Teckman, Andrea Ballabio, and Nicola Brunetti-Pierri.

*Corresponding author: Nicola Brunetti-Pierri, Telethon Institute of Genetics and Medicine*

**Review timeline:**

| | |
|---|---|
| Submission date: | 19 September 2012 |
| Editorial Decision: | 31 October 2012 |
| Revision received: | 02 December 2012 |
| Editorial Decision: | 11 December 2012 |
| Revision received: | 13 December 2012 |
| Accepted: | 15 December 2012 |

## Transaction Report:

(Note: With the exception of the correction of typographical or spelling errors that could be a source of ambiguity, letters and reports are not edited. The original formatting of letters and referee reports may not be reflected in this compilation.)

*Editor: Roberto Buccione*

1st Editorial Decision 31 October 2012

Thank you for the submission of your manuscript to EMBO Molecular Medicine. We have now heard back from the three Reviewers whom we asked to evaluate your manuscript.

All Reviewers are quite positive and find the topic of the study important. They do, however raise a number of concerns about the interpretation of the results and important technical issues, which should be convincingly addressed with further experimentation where necessary, in a revision of the current manuscript. Furthermore, as you will see from the enclosed reports, Reviewers 1 and 3 both express concern on the mouse embryo fibroblast experiments in Figure 1 with respect to the observed increase in mutant ATT protein secretion under TFEB overexpression.

I should remind you that it is EMBO Molecular Medicine policy to allow a single round of revision only and that, therefore, acceptance or rejection of the manuscript will depend on the completeness of your responses included in the next, final version of the manuscript.

Revised manuscripts should be submitted within three months of a request for revision; they will otherwise be treated as new submissions, except under exceptional circumstances in which a short extension is obtained from the editor.

I look forward to seeing a revised form of your manuscript as soon as possible.

***** Reviewer's comments *****

Referee #1 (General Remarks):

Reviewer comments:
The manuscript by Pastore et al., describes an interesting gene transfer strategy for the treatment of AAT-deficiency. The strategy consists in the overexpression of the TFEB transcription factor in the liver that increases autophagy and lysosome biogenesis in the hepatocytes reducing the accumulation of the hepatotoxic ATZ protein (AAT mutant protein). The therapeutic effect is clear and has been very well demonstrated by the authors in a relevant animal model of AAT deficiency. Importantly constitutive expression of TFEB is not toxic and the reduction of ATZ expression levels reduces the risk of HCC development. One interesting aspect of this strategy is that it will be valid for patients with different genetic deficiency leading to AAT accumulation. Furthermore this therapeutic strategy could be apply to other disease associated to the intracellular accumulation of misfolded proteins.

However, some authors have previously reported the use of molecules to increase autophagy for the treatment of AAT deficiency reducing the novelty of the approach presented in this work.

The paper has some general and some specific flaws.

1. The quality of figures 1A and 1B should be improved since it is not clear which are the ATZ bands in each case.

2. The authors are using a long term expression vector, toxicity analysis should be also performed at later time points after vector administration.

3. Since the reduction of intracellular ATZ is associated with an increase of ATZ secretion in which percentage the reduction of ATZ after TFEB expression is due to autophagy or to the increase of protein secretion? Why TFEB expression increase ATZ secretion?

Carbazepine (CBZ) induces ATZ reduction by activation of autophagy but also by proteasome degradation; did the author check if the proteasome play a role on ATZ reduction after TFEB expression?

4. It has been reported that Autophagy abrogation increase ATZ secretion so it will be expected that autophagy activation will reduce ATZ secretion, the authors should comment on this.

5. Does TFEB expression result in ATZ secretion to the circulation?

6. CBZ induce an increase in the number of autophagosomes, however, TFEB expression decrease the number of autophagosomes, the explanation given by the authors is that this is due to fusion of the autophagosomes to the lysosomes, meaning that once the autophagomes are fused to the lysosomes they cannot be detected?

7. It will be interesting to see if TFEB expression induces ATG7, ATG12 and LC3 expression in the liver.

8. The authors should analyse if TFEB expression affects the secretion of AAT and depending on the results they should comment on how lung pathology associated with AAT-deficiency will be affected.

9. Indicate in Fig 2D that the graph represents the fold increase in TFEB expression.

Referee #2 (General Remarks):

The manuscript by Pastore, et al., attempts to confirm the hypothesis that augmentation of autophagy will increase the degradation of Z-mutant alpha-1 antitrypsin (ATZ) both in cell lines and in the transgenic ATZ over-expressing mouse model.

The basis for the experiments is augmentation of transcription factor EB (TFEB). This is done in

cell lines by transfection, which are then assessed by pulse-chase labeling, both in transient transfections and stable cell lines.

Subsequent experiments utilized helper-dependendent Ad vectors (HDAd) to sugment TFEB in the transgenic mouse model. The key finding that Z-AAT accumulation in the liver was reduced was demonstrated convincingly. Other experiments demonstrated a decrease in both monomeric and polymeric ATZ. The changes in ATZ accumulation were correlated with a number of other key findings including an improvment in mitochondrial ultrastructure and a demonstration of autophagy markers being increased.

Finally, there is evidence presented that the HDAd-TFEB augmentation actually decreased the amount of AAT mRNA. This reinforces a concept presented as a model in Figure 8, that the cellular response to the mutant AAT results in increased IL-6-mediated up-regulation of AAT expression.

Referee #3 (Comments on Novelty/Model System):

This is a nice straightforward study that uses a robust model of A1AT liver disease to show that gene trasfer of TFEB prevents mutant A1AT accumulaton, liver injury, and liver fibrosis. The results are very good but expected.

Referee #3 (General Remarks):

This is a nice straightforward study that uses a robust model of AAT liver disease to show that gene trasfer of TFEB prevents mutant AAT accumulaton, liver injury, and liver fibrosis.

1. The MEF data in figure 1 is the least convincing. Why is there increased secretion of A1AT with TFEB treatment? The pulse chase is also difficult to interpret. Why not use primary cultures of hepatocytes from the A1AT Z mouse to demonstrate the effects?

2. Figure 2 is very impressive, but would benefit from a higher magnification of 2A in order to clearly visualize the PAS positive inclusion bodies.

3. Figure 4. The HDAd-TFEB is on different gels than the controls so that comparisons are tenuous. The whole experiment should be run on one gel.

1st Revision - authors' response 02 December 2012

2$^{nd}$ December, 2012

RE: Resubmission of manuscript EMM-2012-02046.

Dear Editor,

Thank you for considering our manuscript entitled "Gene transfer of master autophagy regulator TFEB results in clearance of toxic protein and correction of hepatic disease in alpha-1-antitrypsin deficiency" by Pastore et al. for publication in *EMBO Molecular Medicine*. We appreciated the thoughtful comments of the reviewers and we included the information pertinent to address their concerns. Enclosed please find a revision that responds to the reviewers' comments and concerns. We have highlighted in red the changes we made in the revised manuscript.

Please see below for a detailed response to each reviewer's comments. We have done our best to respond to the concerns of reviewer #1 and #3 (reviewer #2 did not had specific critiques) and have addressed them in point-by-point fashion.

*Reviewer #1 comments:*

*The manuscript by Pastore et al., describes an interesting gene transfer strategy for the treatment of AAT-deficiency. The strategy consists in the overexpression of the TFEB transcription factor in the liver that increases autophagy and lysosome biogenesis in the hepatocytes reducing the accumulation of the hepatotoxic ATZ protein (AAT mutant protein). The therapeutic effect is clear and has been very well demonstrated by the authors in a relevant animal model of AAT deficiency. Importantly constitutive expression of TFEB is not toxic and the reduction of ATZ expression levels reduces the risk of HCC development. One interesting aspect of this strategy is that it will be valid for patients with different genetic deficiency leading to AAT accumulation. Furthermore this therapeutic strategy could be apply to other disease associated to the intracellular accumulation of misfolded proteins. However, some authors have previously reported the use of molecules to increase autophagy for the treatment of AAT deficiency reducing the novelty of the approach presented in this work.*

*The paper has some general and some specific flaws.*

1. *The quality of figures 1A and 1B should be improved since it is not clear which are the ATZ bands in each case.*

   Author's reply: We have repeated the in vitro studies and we are now presenting images of improved quality that show ATZ bands more clearly (revised Fig. 1).

2. *The authors are using a long term expression vector, toxicity analysis should be also performed at later time points after vector administration.*

   Author's reply: We have evaluated toxicity also at later time points (up to 6 months post-injection) in the PiZ mice injected with HDAd-TFEB vector and in controls (Supplementary Figures 4 and 5).

3. *Since the reduction of intracellular ATZ is associated with an increase of ATZ secretion in which percentage the reduction of ATZ after TFEB expression is due to autophagy or to the increase of protein secretion? Why TFEB expression increase ATZ secretion? Carbazepine (CBZ) induces ATZ reduction by activation of autophagy but also by proteasome degradation; did the author check if the proteasome play a role on ATZ reduction after TFEB expression?*

   Author's reply: We have repeated the pulse and chase experiments and performed a careful determination of the ATZ band in the media that was not previously performed. Besides the reduction of intracellular ATZ, upon quantification in media we observe a decreased ATZ secretion in cells transfected with TFEB. We are providing these new data on ATZ secretion in the revised Fig. 1 (please see panels A and C of revised Fig. 1).

   We treated MEFs with MG132 proteasome inhibitor and we observed in TFEB transfected cells a similar reduction in intracellular ATZ compared to untreated cells. Therefore, TFEB does activate proteasome degradation of ATZ in MEF. This new data has been included as panel E in revised Fig. 1.

4. *It has been reported that Autophagy abrogation increase ATZ secretion so it will be expected that autophagy activation will reduce ATZ secretion, the authors should comment on this.*

   Author's reply: We indeed observed a reduction of both intracellular and extracellular ATZ (please see panels A and C of revised Fig. 1). The results of our studies are now consistent with the reported role of autophagy on ATZ secretion.

5. *Does TFEB expression result in ATZ secretion to the circulation?*

   Author's reply: Consistent with the reduction in monomeric ATZ (Fig. 5), we observed a reduction in serum ATZ over time in HDAd-TFEB injected mice. These data have been added as Fig. 3.

6. *CBZ induce an increase in the number of autophagosomes, however, TFEB expression decrease the number of autophagosomes, the explanation given by the authors is that this is due to fusion of the autophagosomes to the lysosomes, meaning that once the autophagomes are fused to the lysosomes they cannot be detected?*

   Author's reply: The reviewer is correct. Upon fusion with the lysosome, autophagosome loses rapidly its specific molecular markers (such as LC3) and its identity as an organelle. Consistent with previously published observations (Teckman et al. Am J Physiol Gastrointest Liver Physiol 279:G961-G974, 2000), we found an increase in autophagosome number in PiZ mice compared to wild-type mice (Fig. 4F). As the reviewer pointed out, HDAd-TFEB injected PiZ mice exhibited less autophagosomes than control PiZ mice, likely because their fusion with lysosomes is activated by TFEB, as previously reported (Settembre et al., Science 332:1429-1433, 2011).

7. *It will be interesting to see if TFEB expression induces ATG7, ATG12 and LC3 expression in the liver.*

   Author's reply: *ATG7* and *ATG12* are not transcriptional targets of TFEB, as previously shown by Settembre et al. (Science 332:1429-1433, 2011, Table 2 of the supplementary

material). We have included LC3 western blot as revised Figure 4 (panel C). As shown by Western blot, LC3 is increased in the liver of HDAd-TFEB injected mice.

8. *The authors should analyse if TFEB expression affects the secretion of AAT and depending on the results they should comment on how lung pathology associated with AAT-deficiency will be affected.*

   Author's reply: We added data on serum levels of AAT (Fig. 3). Moreover, we included a discussion on the lung disease (discussion, page 17).

9. *Indicate in Fig 2D that the graph represents the fold increase in TFEB expression.*

   Author's reply: This information has been included in revised Fig. 2D.

Referee #3 (Comments on Novelty/Model System):

*This is a nice straightforward study that uses a robust model of A1AT liver disease to show that gene transfer of TFEB prevents mutant A1AT accumulation, liver injury, and liver fibrosis. The results are very good but expected.*

Referee #3 (Remarks):

*This is a nice straightforward study that uses a robust model of AAT liver disease to show that gene transfer of TFEB prevents mutant AAT accumulation, liver injury, and liver fibrosis.*

1. *The MEF data in figure 1 is the least convincing. Why is there increased secretion of A1AT with TFEB treatment? The pulse chase is also difficult to interpret. Why not use primary cultures of hepatocytes from the A1AT Z mouse to demonstrate the effects?*

   Author's reply: We found ATZ secretion to be reduced and not increased as shown by the quantification of the pulse and chase in the media (please see reply to issue #3 from reviewer #1 and revised Fig. 1). Experiments with primary hepatocytes, including transfections, are notoriously difficult. Moreover, we elected to use MEFs because we wanted to investigate the role of functioning autophagy in TFEB-mediated clearance of ATZ by comparing wild-type and Atg7-/- MEFs.

2. *Figure 2 is very impressive, but would benefit from a higher magnification of 2A in order to clearly visualize the PAS positive inclusion bodies.*

   Author's reply: We have included figures of PAS staining figures at higher (40X) magnification in the revised fig. 2 and in the supplementary Fig. 3B. The PAS positive inclusion bodies are well visualized with this higher magnification.

3. *Figure 4. The HDAd-TFEB is on different gels than the controls so that comparisons are tenuous. The whole experiment should be run on one gel.*

   Author's reply: The samples corresponding to livers of HDAd-TFEB injected mice were run on the same gel. Similarly to other gels presented in the manuscript, the whole experiment has n=5 samples. Consistently throughout the manuscript, we elected to show 3 out of the 5 samples for each experimental group. We are submitting the whole gel as an extra file to

show that samples were run all together. However, for consistency with other experiments presented in the manuscript, we would prefer to show 3 out of the 5 samples.

Thank you again for your encouraging response and we hope the revised version of this manuscript may now be suitable for publication in a future issue of *EMBO Molecular Medicine*.

2nd Editorial Decision 11 December 2012

Thank you for the submission of your revised manuscript to EMBO Molecular Medicine. We have now received the enclosed report from the Reviewer 1 who was asked to re-assess it. As you will see the Reviewer is generally satisfied but raises a specific concern based on your revised version, with respect to the data on ATZ secretion.

I acknowledge that you mention and briefly discuss the discrepancy in your point-by-point response, but I must agree with the Reviewer that the new data require a better explanation.

Please submit your revised manuscript within two weeks and possibly before the holiday break.

I look forward to reading a new revised version of your manuscript as soon as possible.

***** Reviewer's comments *****

Referee #1 (General Remarks):

The authors have properly addressed all the comments; however, I found strange the new data on ATZ secretion. In the previous version of the manuscript the authors reported an increase in protein secretion in TFEB transfected WT fibroblast and also an increase secretion in Atg7-/- fibroblasts. However, in the current version of the manuscript the results are the opposite: there is a decrease in ATZ secretion after TFEB transduction and no differences between WT and Atg7-7- fibroblasts. Furthermore, the densitometric values presented in the first version of the paper are quite different from the data presented in the current version deserving a clear explanation for these discrepancies.

2nd Revision - authors' response 13 December 2012

13th December, 2012

RE: manuscript EMM-2012-02046-V2.

Dear Editor,

Please find below our response to the reviewer #1 c oncern on our revised manuscript entitled "Gene transfer of master autophagy regulator TFEB results in clearance of toxic protein and correction of hepatic disease in alpha-1-antitrypsin deficiency" by Pastore et al.

*The authors have properly addressed all the comments; however, I found strange the new data on ATZ secretion. In the previous version of the manuscript the authors reported an increase in protein secretion in TFEB transfected WT fibroblast and also an increase secretion in Atg7-/- fibroblasts. However, in the current version of the manuscript the results are the opposite: there is a decrease in ATZ secretion after TFEB transduction and no differences between WT and Atg7-7- fibroblasts. Furthermore, the densitometric values presented in the first version of the paper are quite different from the data presented in the current version deserving a clear explanation for these discrepancies.*

Author's response: In the first submission of the manuscript, we did not evaluated by densitometry the intensities of the bands corresponding to ATZ in the media (please see fig. 1 from the first submission). This first experiment was performed with a chase of 3 hours and the bands corresponding to ATZ in the media suggested us an increased ATZ secretion. As requested by the reviewers, we repeated the experiment and to address their specific questions on ATZ secretion, we performed an accurate quantification of the secreted protein, in addition to the intracellular ATZ. In contrast with the previous experiment with 3 hours of chase, this repeated experiment was performed for a longer period of chasing of 5 hours. Upon quantification of ATZ bands in media on this repeated experiment, we did not confirm the increased ATZ secretion and we detected reduced ATZ secretion particularly after 3 hours of chase. We attributed this different results, at least in part, to the shorter period of chase and to the lack of quantification on media. Nevertheless, because of this discrepancy, we repeated the pulse and chase experiment one more time and we confirmed the result obtained in the second experiment with 5 hour chase that has been presented in the revised manuscript. We are submitting as an extra-file the repeated pulse and chase experiment that confirmed the results (Figure 1 of the extra file).

The comparison between WT and Atg7-/- fibroblasts included in the revised Fig.1 cannot be performed because the gel included in panel B (Atg7-/- MEF) of the Fig. 1 were not exposed for the same time of the gel in panel A (w.t. MEF). We have gels from Atg7-/- and w.t. MEFs that were exposed at the same time and on these gels. On these gels, we detected a mild increase in ATZ secretion in Atg7-/- cells that reached statistical significance only at one time point. These gels are also shown in the extra file (Figure 2 of the extra file). Nevertheless, in these experiments we are looking at the effect of TFEB on ATZ clearance in w.t. and autophagy-deficient cells and not at the effect of deficient autophagy on ATZ, that has been already shown in previous publications. For this reason, the gels presented in panels A and B were not exposed together.

The densitometric values of the intracellular ATZ (the media ATZ was not quantified in the first experiment) up to 3 hours are similar between the two experiments: the intracellular ATZ in the first experiment was 53.8±6% of the baseline at 3 hours while it was 40.3±9.1% at the same time point of the repeated experiment, in TFEB transfected cells; the intracellular ATZ in the first experiment was 106.5±12% of the baseline at 3 hours while it was 70±3.6% at the same time point in the repeated experiment, in GFP transfected cells. In Atg7-/- cells, intracellular ATZ was 121±41.3% of baseline at 3 hours in the first experiment and 70.4±28.8% of baseline at the same time point of the repeated experiment, in TFEB transfected cells; the intracellular ATZ in the first experiment was 82.1±33.3% of the baseline at 3 hours, while it was 93.4±3.7% of baseline at the same time point of the repeated experiment, in GFP transfected cells.

We hope this response will satisfy both you and the reviewer.
