# Supplementary material for: Gene transfer of master autophagy regulator TFEB results in clearance of toxic protein and correction of hepatic disease in alpha-1-anti-trypsin deficiency
Source: EMBO Mol Med. 2013 Feb 4;5(3):397–412. doi: 10.1002/emmm.201202046 (PMC3598080; doi:10.1002/emmm.201202046)
Supplement: Supplementary file 2 [file emmm0005-0397-SD2.pdf]

Supplementary Material

Title: Gene transfer of master autophagy regulator TFEB results in clearance of toxic protein and correction of hepatic disease in alpha-1-antitrypsin deficiency

Authors: Nunzia Pastore[1], Keith Blomenkamp[2], Fabio Annunziata[1], Pasquale Piccolo[1], Pratibha Mithbaokar[1], Rosa Maria Sepe[1], Francesco Vetrini[3], Donna Palmer[3], Philip Ng[3], Elena Polishchuk[1], Simona Iacobacci[1], Roman Polishchuk[1], Jeffrey Teckman[2], Andrea Ballabio[1,3,4,5], and Nicola Brunetti-Pierri[1,5,*].

Affiliations: [1]Telethon Institute of Genetics and Medicine, Naples, Italy; [2]Department of Pediatrics, Saint Louis University School of Medicine, Cardinal Glennon Children's Medical Center, Saint Louis, MO, USA; [3]Department of Molecular and Human Genetics, Baylor College of Medicine, Houston, TX, USA; [4]Jan and Dan Duncan Neurological Research Institute, Texas Children’s Hospital, Houston, TX, USA; [5]Department of Pediatrics, Federico II University, Naples, Italy.

Table of contents

Supplementary Fig. 1. HDAd-TFEB vector.

Supplementary Fig. 2. Body weight curves.

Supplementary Fig. 3. ATZ Western blot in injected PiZ mice and PAS staining in untreated PiZ controls.

Supplementary Fig. 4. AST and ALT.

Supplementary Fig. 5. Expression of hepatocyte-specific genes.

Supplementary Fig. 6. Mitochondria in HDAd-TFEB injected mice.

Supplementary Fig. 7. TFEB gene transfer has no effect on NFκB in wild-type mice.

Supplementary Fig. 1. HDAd-TFEB vector.

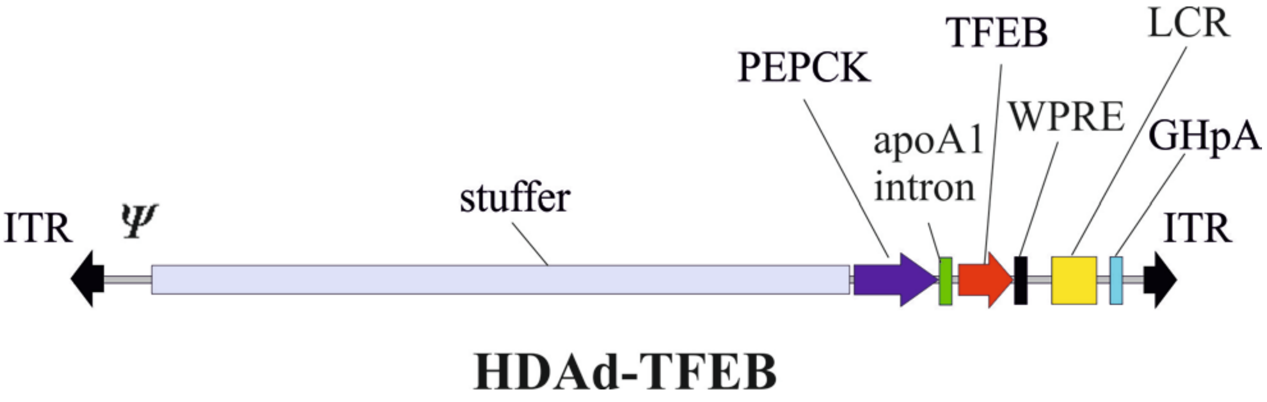

HDAd-TFEB contained the human TFEB transgene under the control of a liver-specific PEPCK promoter. The expression cassette included the ApoAI intron, the woodchuck hepatitis post-transcriptional regulatory element (WPRE), the Locus Control Region (LCR) from the apoE locus and the human growth hormone poly A (GHpA). Adenoviral inverted terminal repeats (ITR) and packaging signal (Ψ) are shown. The HDAd-AFP vector also used in this study is identical to this vector with the exception of the transgene that is the baboon alpha-fetoprotein (AFP) gene. Not drawn to scale.

Supplementary Fig. 2. Body weight curves.

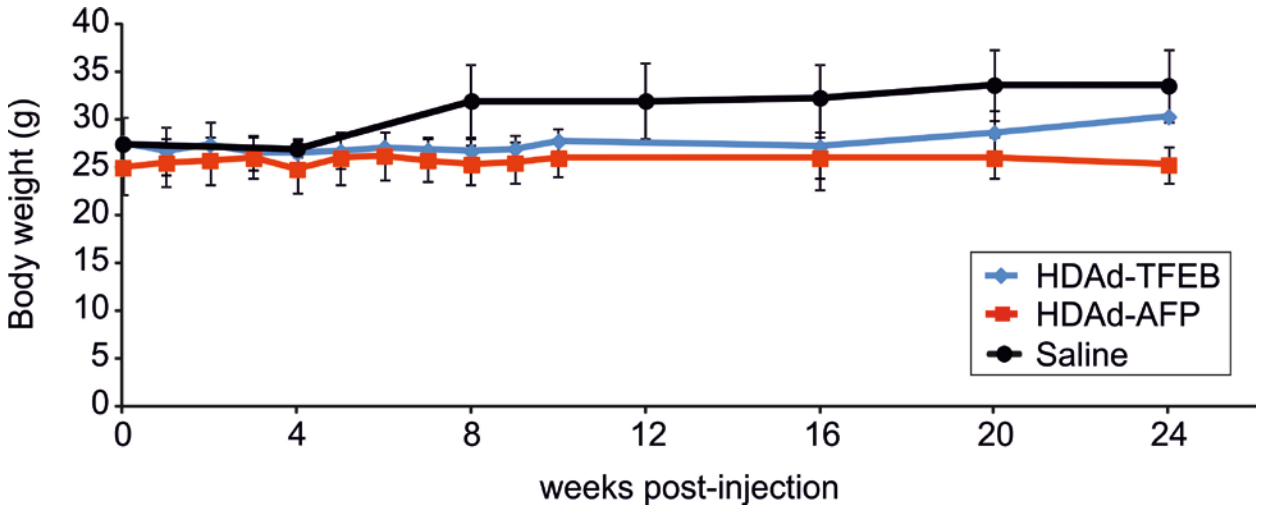

Body weights did not show significant differences between HDAd-TFEB, HDAd-AFP, and saline injected mice for up to 6 months post-injection (at least n=5 per group). Averages ± standard deviations are shown.

Supplementary Fig. 3. ATZ Western blot in injected PiZ mice and PAS staining in untreated PiZ controls.

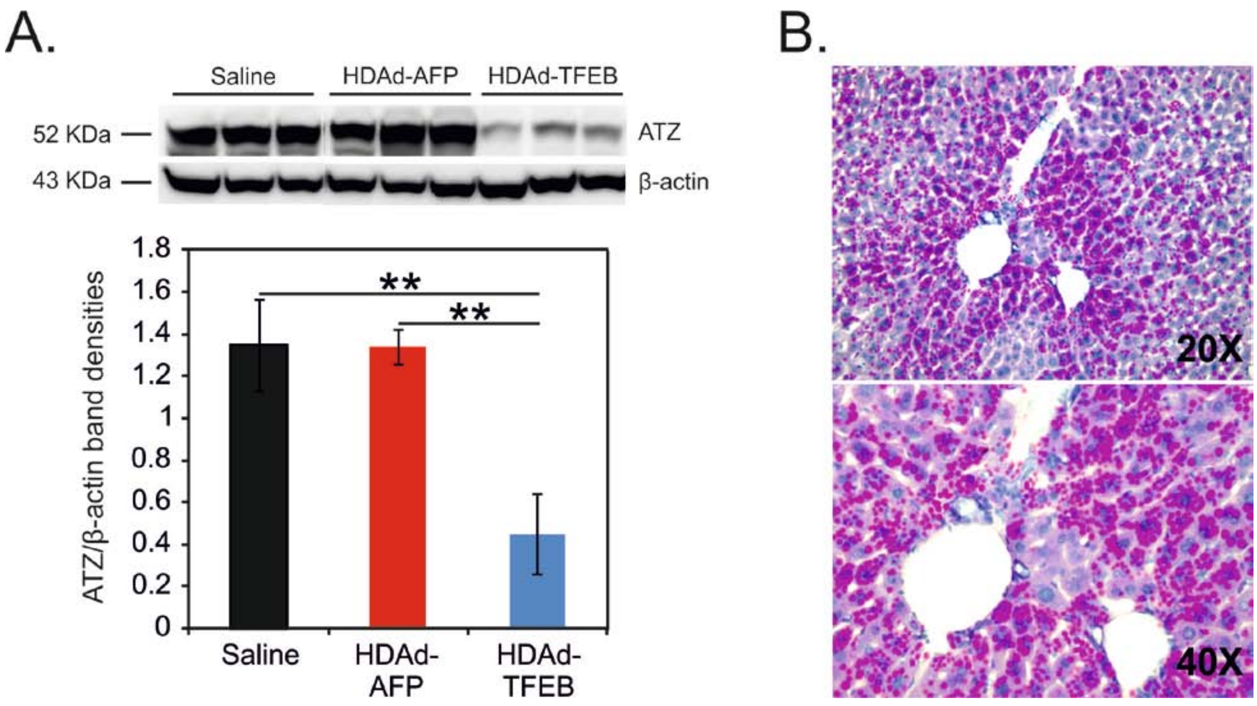

(**A**) Western blot analysis showed a significant reduction in ATZ band intensities in livers of HDAd-TFEB injected PiZ mice compared to HDAd-AFP and saline injected controls. Three of five representative mice are shown in the western blot analysis. Quantification of band intensities was performed on n=5 mice per group. Averages ± standard deviations are shown. $^{**}p<0.01$. (**B**) Representative PAS staining of livers from PiZ mice of 3 months of age that corresponds to the age of PiZ mice injected with HDAd-TFEB, HDAd-AFP, or saline included in **Fig. 2** (magnifications 20X and 40X).

Supplementary Fig. 4. AST and ALT.

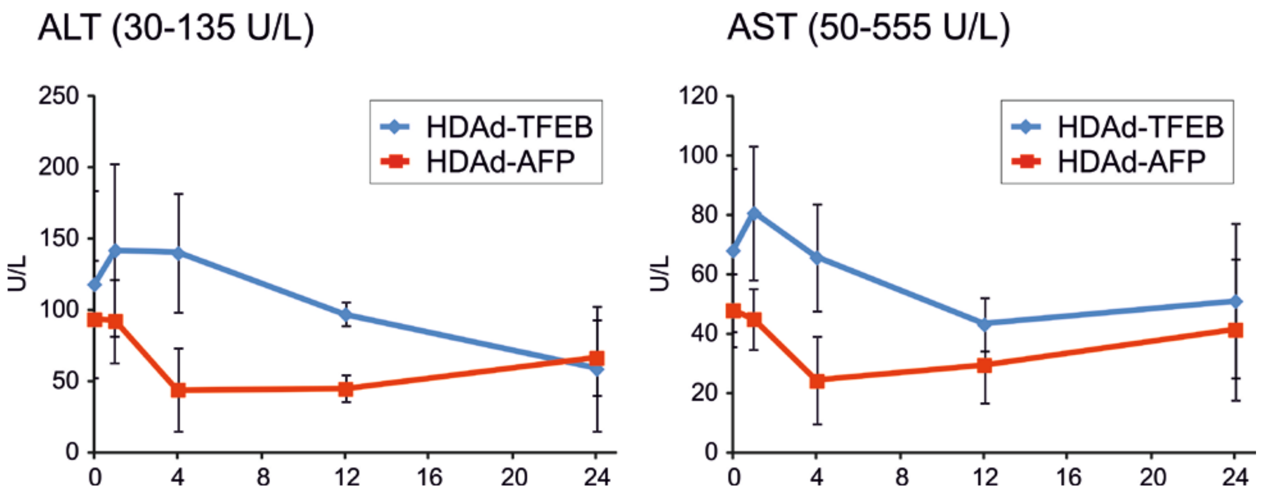

AST and ALT remained within the normal ranges and no significant differences were noted between HDAd-TFEB and HDAd-AFP injected mice (n=5 per group). Averages ± standard deviations are shown. Normal ranges in parenthesis are based on normal ranges determined for C57BL/6 mice (Schnell et al, 2002).

Schnell MA, Hardy C, Hawley M, Propert KJ, Wilson JM (2002) Effect of blood collection technique in mice on clinical pathology parameters. Hum Gene Ther 13**:** 155-161.

Supplementary Fig. 5. Expression of hepatocyte-specific genes.

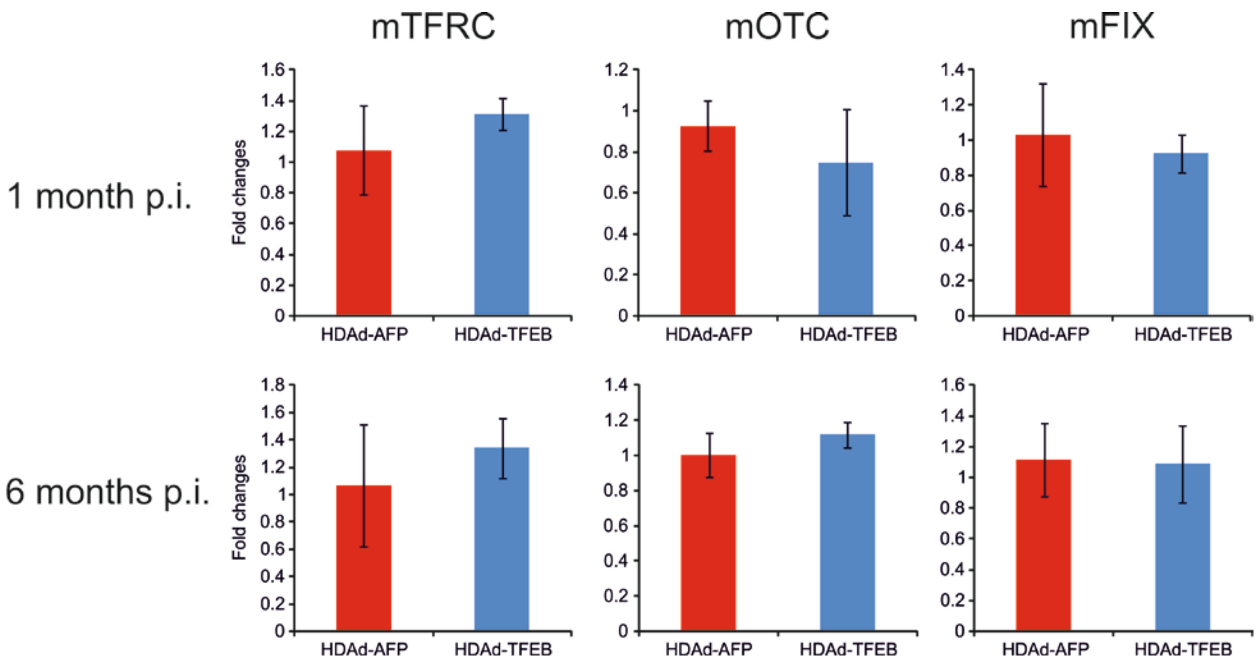

No significant differences were detected in mouse hepatocyte specific mRNA of transferrin receptor (mTFRC), ornithine carbamoyltransferase (mOTC), and factor IX (mFIX) between HDAd-TFEB and HDAd-AFP injected mice (n=5 per group). Averages ± standard deviations are shown. Abbreviation: p.i.= post-injection.

Supplementary Fig. 6. Mitochondria in HDAd-TFEB injected mice.

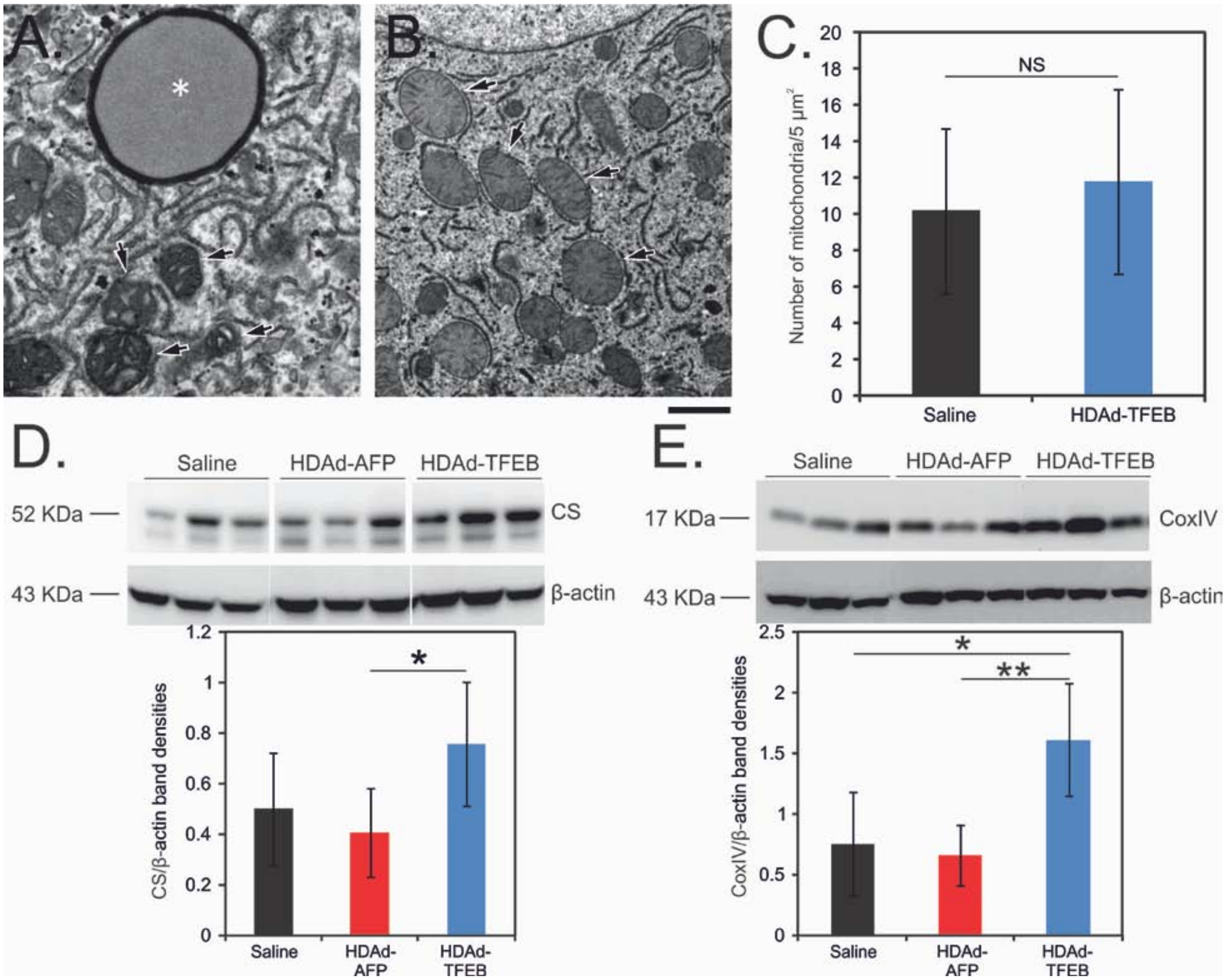

Ultrastructure of mitochondria in saline (**A**) and TFEB-injected (**B**) PiZ mice. (A) Hepatocytes with inclusions (asterisk) in saline-treated frequently contain mitochondria with swollen cristae (arrows). (**B**) Mitochondria (arrows) in HDAd-TFEB injected mice exhibited regular ultrastructure with thin cristae. Scale bar, 450 nm (**A, B**). (**C**) Number of mitochondria was measured by EM and no significant differences were detected between saline and HDAd-TFEB-injected mice. (**D**) Mitochondrial citrate synthase (CS) was increased in HDAd-TFEB injected mice compared to HDAd-AFP injected mice. (**E**) Mitochondrial CoxIV protein was increased in HDAd-TFEB injected mice compared to HDAd-AFP or saline injected mice. For **D** and **E**, representative bands from 3 mice for each treatment group are shown. The graph shows densitometric quantification of n=5 mice from each treatment group. Average ± standard deviation is shown. NS= not statistical significant difference. $^{**}p<0.01$; $^{*}p<0.05$.

Supplementary Fig. 7. TFEB gene transfer has no effect on NFκB in wild-type mice.

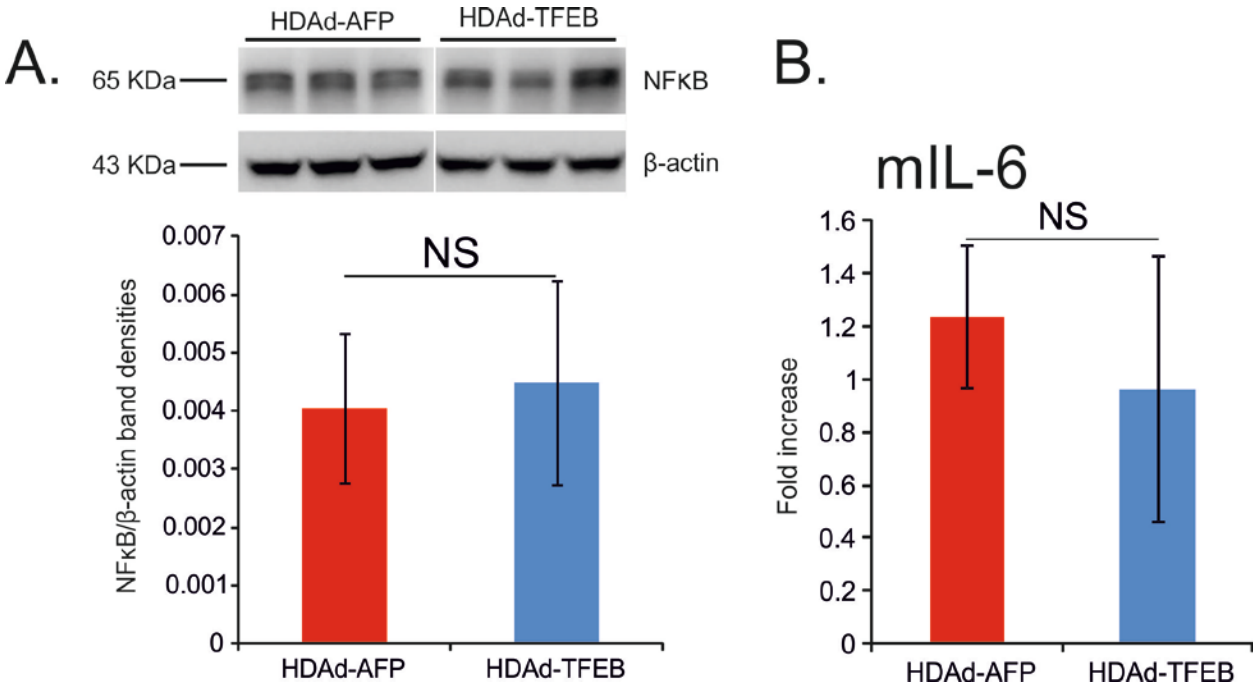

Liver-directed TFEB gene transfer in C56BL/6 wild-type mice had no effect on hepatic NFκB protein levels (**A**) or mIL-6 mRNA (**B**). Representative bands from 3 mice for each treatment group are shown. The graph shows densitometric quantification of n=5 mice from each treatment group. The mIL-6 was measured by real time PCR in mRNA extracted from livers of injected animals (n=5 per group). Averages ± standard deviations are shown. NS= not statistical significant difference.
